# L-fucose administration ameliorates chronic social defeat stress-induced depressive-like behaviors by restoring GDP-fucose biosynthetic activation and core fucosylation

**DOI:** 10.3389/fimmu.2026.1909378

**Published:** 2026-07-30

**Authors:** Meng Zheng, Tomohiko Fukuda, Siming Zhang, Yue Wang, Tsukushi Saito, Yuhang Zhou, Boyu Xia, Kazuki Mochizuki, Teruki Aizawa, Tomoya Isaji, Jianguo Gu

**Affiliations:** 1 Division of Regulatory Glycobiology, Graduate School of Pharmaceutical Sciences, Tohoku Medical and Pharmaceutical University, Sendai, Japan; 2 Institute of Molecular Biomembrane and Glycobiology, Tohoku Medical and Pharmaceutical University, Sendai, Japan; 3 Yaizu Suisankagaku Industry Co., Ltd., Yaizu, Japan

**Keywords:** chronic social defeat stress, core fucosylation, depression, GDP-fucose, neuroinflammation

## Abstract

Chronic psychosocial stress is a major driver of depression, yet the glycometabolic mechanisms linking stress exposure to neuroinflammation and synaptic dysfunction remain poorly understood. Here, using a chronic social defeat stress (CSDS) model, we demonstrated that L-fucose supplementation markedly attenuated depressive-like behaviors and suppressed neuroinflammation in the hippocampus. L-fucose also reduced Iba1-positive microglial accumulation, inhibited JAK2/STAT3 inflammatory signaling, and restored the stress-induced loss of synaptic proteins, including α-amino-3-hydroxy-5-methyl-4-isoxazolepropionic acid (AMPA) receptor subunits and postsynaptic density protein 95 (PSD95). Mechanistically, CSDS downregulated hippocampal guanosine 5′-diphosphate (GDP)-fucose levels, thereby impairing core fucosylation. This defect was associated with downregulation of key enzymes in the GDP-fucose salvage biosynthetic pathway, including fucokinase (FUK) and fucose-1-phosphate guanylyltransferase (FPGT), both of which were restored by L-fucose administration. Consistently, pharmacological inhibition of fucosylation with 2-fluorofucose (2FF) suppressed GDP-fucose and *Fuk* expression, thereby exacerbating depressive-like behaviors and neuroinflammation in the CSDS model. Furthermore, L-fucose or MF30 alleviated CSDS-induced inflammatory responses in distal colon tissues. Taken together, this study clearly identifies a link between GDP-fucose synthesis and chronic stress and, for the first time, shows that alterations in key enzymes in the GDP-fucose salvage pathway play critical roles in CSDS-induced depressive-like behaviors and neuroinflammation, suggesting that restoring GDP-fucose biosynthetic activation may offer a promising therapeutic strategy for stress-related neuropsychiatric disorders.

## Introduction

Depression is a stress-related neuropsychiatric disorder characterized by persistent affective disturbance, cognitive impairment, and increased vulnerability to relapse (1, 2). Converging clinical and experimental evidence indicates that chronic stress drives sustained neuroinflammatory signaling in the brain (3, 4). Elevated levels of pro-inflammatory cytokines, including interleukin-6 (IL-6), interleukin-1β (IL-1β), and tumor necrosis factor-α (TNF-α), have been consistently reported in patients with depression and in rodent stress models (5, 6). Microglia, the resident immune cells of the central nervous system (CNS), are highly responsive to stress and can adopt a pro-inflammatory phenotype characterized by excessive cytokine production and altered synaptic remodeling (7, 8). By sustaining local inflammatory signaling, activated microglia release inflammatory mediators that impair synaptic plasticity and excitatory neurotransmission, thereby contributing to depressive-like behaviors (9–11). Prolonged inflammatory activation further promotes neuronal dysfunction and structural abnormalities in stress-sensitive brain regions such as the hippocampus, which plays a central role in mood regulation and synaptic plasticity (12–14).

It is well known that social stress-related psychiatric disorders have become increasingly prevalent amid modern socioeconomic and interpersonal pressures. Importantly, chronic psychosocial stress is considered a major environmental risk factor for depression in modern society (15, 16). Social isolation, interpersonal conflict, workplace pressure, and repeated social adversity have been strongly associated with increased susceptibility to depressive disorders in humans (15–17). To experimentally model these stress-related pathological processes, chronic social defeat stress (CSDS) has been widely used as a clinically relevant rodent model that recapitulates persistent social avoidance, anhedonia, neuroinflammatory activation, and stress vulnerability induced by repeated social stress exposure (18, 19). Unlike conventional non-social stress paradigms such as chronic unpredictable stress (CUS), which rely on heterogeneous physical and environmental stressors, CSDS specifically models repeated psychosocial stress exposure and more closely reflects social adversity commonly experienced in human society. Moreover, CSDS exhibits robust behavioral stratification and high translational relevance to stress-associated depression (19, 20). These findings support a close relationship between chronic stress, neuroinflammation, and synaptic dysfunction in depression. However, whether chronic stress directly alters metabolic pathways required for neuroimmune and synaptic homeostasis remains unclear.

Recent evidence suggests that metabolic regulation of glycosylation critically influences inflammatory signaling and neuronal function in the CNS (21–23). Among the diverse glycosylation modifications, fucosylation has emerged as an important regulator of receptor-mediated signaling and immune responses (24, 25). Altered glycosylation can influence receptor stability, ligand affinity, membrane trafficking, and downstream inflammatory signaling, processes that critically regulate immune activation and neuronal communication within the CNS (22, 26). Accumulating evidence further indicates that L-fucose exerts anti-inflammatory effects in multiple pathological conditions (27, 28), including neuroinflammation (29) and Alzheimer’s disease models (30). L-fucose can be metabolized to guanosine 5′-diphosphate-fucose (GDP-fucose), a donor substrate for fucosyltransferase-catalyzed glycosylation (24, 31, 32), via a fucokinase (FUK) and fucose-1-phosphate guanylyltransferase (FPGT)-dependent salvage pathway. Intracellular GDP-fucose is synthesized via two pathways: a *de novo* pathway and a salvage pathway (33). The *de novo* pathway generates GDP-fucose from GDP-mannose through GDP-mannose 4,6-dehydratase (GMDS) and GDP-L-fucose synthase (TSTA3/FX), whereas the salvage pathway converts free L-fucose to fucose-1-phosphate through FUK, followed by conversion to GDP-fucose via FPGT. While the *de novo* pathway maintains basal GDP-fucose synthesis, the salvage pathway reutilizes free L-fucose to replenish intracellular GDP-fucose pools under changing physiological or pathological conditions (24, 32, 34). In particular, the salvage pathway is more metabolically responsive and can rapidly adjust intracellular GDP-fucose availability in response to metabolic demand or environmental stress (31, 35). Consequently, alterations in L-fucose availability or salvage pathway activity may substantially influence intracellular GDP-fucose homeostasis and downstream fucosylation-dependent signaling. Among various forms of fucosylation, core fucosylation—defined by α1,6-linked fucose addition to the innermost N-acetylglucosamine residue of N-glycans—is catalyzed exclusively by α1,6-fucosyltransferase (Fut8) (25, 36). Core fucosylation has been shown to regulate receptor stability, ligand-binding affinity, membrane localization, and downstream signaling in multiple receptor systems, including cytokine receptor complexes (32, 37).

Our recent studies have demonstrated that Fut8-mediated core fucosylation negatively regulates neuroinflammatory signaling and microglial activation, and that L-fucose supplementation attenuates neuroinflammatory responses and preserves synaptic integrity in lipopolysaccharide (LPS)- and CUS-induced depression models (29, 38, 39). Based on these observations, we hypothesized that intracellular fucose metabolism serves as a stress-responsive regulatory pathway linking neuroimmune activation to synaptic dysfunction in depression. In the present study, we used the CSDS model, which closely mimics human social stress-related depression, to determine whether chronic psychosocial stress disrupts hippocampal GDP-fucose biosynthesis pathways, particularly the salvage pathway, and whether restoring fucose metabolism with L-fucose could alleviate neuroinflammatory and behavioral abnormalities. Our findings first identify impaired GDP-fucose biosynthesis, rather than changes in Fut8 expression, as a plausible mechanism in chronic stress-induced depression, suggesting that restoring fucose metabolic activation may be a therapeutic strategy for stress-related neuropsychiatric disorders.

## Methods

### Antibodies and reagents

The following antibodies and reagents were used: Biotinylated Lens culinaris agglutinin (LCA; J207), which preferentially recognizes core fucosylation, was obtained from J-Oil Mills (Tokyo, Japan). The anti-GAPDH antibody (G9545) and anti-mouse IgG antibody (AP124P) were obtained from Sigma. The ABC kit (PK-4000) was from Vector Laboratories. Anti-Lewis X (A2578), L-fucose (F0065), and 2-fluorofucose (2FF) were purchased from TCI and Synchem, Inc. (IL, USA), respectively. Anti-JAK2 (3230), anti-Phospho-JAK2 (3771), anti-STAT3 (9139), anti-Phospho-STAT3 (9145), anti-GluR1 (13185), and HRP-linked anti-rabbit IgG (7074) antibodies were obtained from Cell Signaling Technology. Anti-PSD95 (04–1066) and anti-GluR2/3 (07–598) antibodies were obtained from Millipore. The anti-Iba1 antibody (019–19741) and 4′,6-diamidino-2-phenylindole dihydrochloride *n*-hydrate (DAPI) were obtained from Wako (Japan). The anti-Fut8 antibody (sc-271244) was obtained from Santa Cruz Biotechnology. The goat anti-rabbit Alexa Fluor 488 antibody (A-11008) was obtained from Invitrogen. Marine Fucose™ 30 (MF30) was kindly provided by Yaizu Suisankagaku Industry Co., Ltd. (Shizuoka, Japan). According to the manufacturer’s information, MF30 is a hydrolyzed seaweed powder containing 50-70% dextrin and 30-50% hydrolyzed seaweed, with an L-fucose content of 33.2%, as determined by HPLC analysis.

### Animals

*Fut8*^+/−^ heterozygous mice on a C57BL/6J genetic background were intercrossed to generate *Fut8*^+/+^ littermates and *Fut8*^+/−^ mice (37). Genotypes were determined by PCR analysis of genomic DNA. All experiments were performed using male mice aged 6–7 weeks (25–30 g). In addition, CD1 mice (8–10 weeks old) were used as aggressors. To enhance territorial aggression, one male and one female CD1 mouse were housed together in a single cage. Prior to each defeat session, the female mouse was removed, and resident male CD1 mice consistently displayed aggressive behavior toward introduced C57BL/6J intruders (40). Mice were housed in groups under standard vivarium conditions with ad libitum access to food and water, a 12-h light/dark cycle (lights on from 07:00 to 19:00), an ambient temperature of 22 ± 2 °C, and a relative humidity of 55 ± 5%. All animal procedures were approved by the Animal Care and Use Committee of the Graduate School of Pharmaceutical Sciences, Tohoku Medical and Pharmaceutical University.

### Experimental design

After genotyping, male *Fut8^+/+^* and *Fut8^+/−^* mice were randomly assigned to experimental groups. *Fut8^+/+^* and *Fut8^+/−^* denote wild-type and heterozygous mice, respectively, and these designations are used consistently throughout the manuscript. Investigators were blinded to treatment and genotype during behavioral scoring and quantitative analyses whenever possible. Animals were excluded only for severe injury, illness, technical failure, or tail climbing during the TST. For the preventive paradigm, *Fut8^+/+^* mice received vehicle or L-fucose by oral gavage twice daily from Day 0 to Day 14 at total doses of 4, 12, or 36 mg/day, while CSDS was performed from Day 1 to Day 10. For the post-CSDS treatment paradigm, *Fut8^+/+^* and *Fut8^+/−^* mice were first subjected to CSDS from Day 1 to Day 10, and then received vehicle or L-fucose at 4 mg/day twice daily from Day 11 to Day 24. MF30 (4 mg/day) was administered by oral gavage using the same post-CSDS treatment schedule. For the 2FF experiment, *Fut8^+/+^* mice received 2FF intraperitoneally at 150 mg/kg/day beginning 3 days before CSDS and continuing throughout the 10-day CSDS period. Behavioral tests were performed on the same cohort of mice in the order shown in the experimental timelines. Unless otherwise indicated, behavioral experiments were performed with n = 6–8 mice per group, and molecular, biochemical, and histological analyses were performed with n = 3–4 mice per group, as indicated in the figure legends.

### Chronic social defeat stress

CSDS was performed as previously described, with minor modifications (41). Retired breeder CD1 male mice were screened for aggressive behavior for 3 consecutive days before experiments. Only mice that initiated an attack within 30 s were selected as aggressors. Experimental mice (*Fut8*^+/−^ or *Fut8*^+/+^) were introduced into the home cage of a novel CD1 aggressor for 5–10 min of direct physical interaction each day for 10 consecutive days. After each session, mice were separated by a perforated transparent divider, allowing sensory contact for the remaining 24 h. Control mice were housed in pairs with perforated dividers but without physical contact. Behavioral testing and tissue collection were performed according to the experimental timelines described above.

### Social interaction test

The SIT was performed as previously reported (41, 42). The test consisted of two 5-min trials conducted in an open-field arena containing a perforated plastic enclosure positioned in the interaction zone. In the first trial (target absent), mice were allowed to freely explore the arena with an empty enclosure. In the second trial (target present), an unfamiliar CD1 mouse was placed inside the enclosure. Time spent in the interaction zone was recorded and analyzed. The social interaction ratio was calculated as the time spent in the interaction zone with a social target divided by the time spent in the interaction zone without the target. The apparatus was cleaned with 70% ethanol between trials.

### Tail suspension test

The TST was conducted as previously described (41, 42). Mice were suspended by the tail with adhesive tape placed approximately 1 cm from the tip, at a height of 50 cm above the floor, for a total of 6 min. Immobility time was measured during the final 4 min of the test. Immobility was defined as remaining completely motionless without active escape-oriented movements. Mice that climbed up their tails during the procedure were excluded from the analysis.

### Forced swimming test

The FST was conducted according to previous studies (41, 42). Mice were individually placed in a transparent glass cylinder (25 cm in height and 10 cm in diameter) filled with water (10 cm depth, 25 ± 1 °C) for 6 min. Immobility was defined as floating without active struggling, with only the minimal movements necessary to keep the head above the water. Immobility time during the final 4 min of the test was recorded and analyzed.

### Sucrose preference test

The SPT was conducted using a previously reported protocol (43). Mice were housed individually and presented with two bottles containing 1% sucrose solution or water. Animals were habituated to the two-bottle choice paradigm for 2 days, with bottle positions switched every 6 h to prevent side preference. After 24 h of food and water deprivation, mice had free access to both bottles for 12 h, after which the bottle positions were exchanged. The volumes of water and sucrose solution consumed were measured. Sucrose preference was calculated as the percentage of sucrose solution intake relative to total fluid intake. All behavioral analyses were performed by investigators blinded to the experimental groups.

### HPLC analysis of nucleotide sugars

Nucleotide sugars were extracted and purified from brain tissues using previously reported procedures (29, 44, 45). For tissue samples, approximately 50 mg of hippocampal tissue was homogenized in 1 mL of 70% ethanol at low temperature using a homogenizer (4500 rpm, 120 s, 2 cycles) and then centrifuged at 15,000 rpm for 10 min at 4 °C to collect the supernatant. Supernatants were frozen and lyophilized. Dried samples were reconstituted in 1 mL of 10 mM ammonium bicarbonate and purified by solid-phase extraction using an ENVI-Carb cartridge (250 mg). The cartridge was conditioned with 2 mL of 80% acetonitrile containing 0.1% trifluoroacetic acid and equilibrated with 1 mL of Milli-Q water. After sample loading, the column was sequentially washed with Milli-Q water (1 mL), 25% acetonitrile (1 mL), Milli-Q water (2 mL), and 50 mM triethylamine acetate (TEAA, pH 7.0, 1 mL). Nucleotide sugars were eluted with 1 mL of 25% acetonitrile in 50 mM TEAA (pH 7.0), and the eluates were lyophilized again. For analysis, lyophilized samples were dissolved in 200 μL of Milli-Q water, and 10 μL was injected for ion-pair reverse-phase HPLC. Detection was performed at 254 nm using an ODS-4 column (4.6 × 250 mm) equipped with a guard column and maintained at 40 °C. The mobile phase consisted of Buffer A (200 mM TEAA, pH 6.0) and Buffer B (70% Buffer A and 30% acetonitrile). The gradient was programmed as follows: 100% Buffer A for 35 min, linear increase to 77% Buffer B over 40 min, increase to 100% Buffer B over 1 min, followed by 100% Buffer B for 14 min. Chromatographic peaks were integrated and quantified using external calibration curves.

### Western blotting and lectin blotting

Equal amounts of protein (10 μg) were resolved under reducing conditions on 7.5% or 10% SDS–PAGE gels at 100 V and transferred to PVDF membranes (Millipore Sigma) using a semi-dry transfer system at 10 V for 1 h. For Western blotting, membranes were blocked with 5% nonfat dry milk in TBST at room temperature for 1.5 h, then incubated with the indicated primary antibodies overnight at 4 °C. For lectin blotting, membranes were blocked with 5% bovine serum albumin (BSA) and incubated with biotinylated LCA. After washing three times with TBST for 10 min each, membranes were incubated with the appropriate HRP-conjugated secondary antibodies for 1.5 h at room temperature. After washing, membranes were incubated with the Vectastain ABC kit (Vector Laboratories) for 1.5 h. Immunoreactive signals were detected using enhanced chemiluminescence (ECL) reagents (Amersham).

### Quantitative PCR

Total RNA was extracted using TRIzol reagent (Invitrogen) according to the manufacturer’s instructions. RNA purity and concentration were determined by spectrophotometry. One microgram of total RNA was reverse-transcribed into cDNA using PrimeScript RT reagent with gDNA Eraser (Takara). qPCR was performed with TB Green^®^ Premix Ex Taq™ II (Tli RNaseH Plus) (Takara) on an Applied Biosystems 7500 qPCR system under the following conditions: initial denaturation at 95 °C for 30 s, followed by 40 cycles of 95 °C for 5 s and 60 °C for 30 s. Relative mRNA expression levels were normalized to glyceraldehyde-3-phosphate dehydrogenase (*Gapdh*) and calculated using the 2^−ΔΔCt^ method. Each biological sample was analyzed in technical triplicate, and amplification specificity was confirmed by melt curve analysis. Primer sequences are listed in **Table 1**.

**Table 1:** Primer sequences for qPCR.

Target genes	Primer sequences (5′-3′)	
	Forward sequences	Reverse sequences
*Il6*	CTGCAAGAGACTTCCATCCAG	AGTGGTATAGACAGGTCTGTTGG
*Tnfα*	AAGTCAACCTCCTCTCTGCC	CCGGACTCCGCAAAGTCTAA
*Il1β*	TGTCTGAAGCAGCTATGGC	TGTTGATGTGCTGCTGCG
*iNOS*	ATGACTCCCAGCACAAAGGG	AACAGCACTCTCTTGCGGACC
*Iba1*	TCTGAGGAGCTATGAGCC	TGCTGTCATTAGAAGGTCC
*Mrc1*	TGGACGCTCTAAGTGCCATC	TCTGCTCCACAATCCCGAAC
*Gapdh*	GGCCTTCCGTGTTCCTAC	TGTCATCATATCTGGCAGGTT
*Fut8*	AACAGAAGCAGCCTTCCACC	TACCGATTGTGTAGTCCAGC
*Gmds*	GCTCAAGCTCCCGCTAAGTG	GTCCGGTGATGCCAGTGAT
*Tsta3*	ATTTCTGGCGGAAAAACGTGC	TGTCTCGTCAATAGGATAGGTGG
*Fuk*	GAGACTTCCCCTTCGATGACT	AGCCATCCCAACTGATCCCA
*Fpgt*	TCTGAAGGTCCTGCTAATCCA	ACCAAGTGGTAAGGCTGTGAA
*Slc35c1*	TGGCTGGGTATAGACCAAGAA	GCACGCAGGCATTGACATTG

### Immunofluorescence staining

After behavioral testing, mice were deeply anesthetized with sodium pentobarbital and transcardially perfused with 50 mL of saline followed by 50 mL of 4% paraformaldehyde. Brains were post-fixed in 4% paraformaldehyde for 24–48 h at 4 °C and dehydrated sequentially in 10%, 20%, and 30% sucrose solutions. Coronal sections (25 μm) containing the hippocampus were prepared on a cryostat and collected in PBS. Sections were permeabilized with 0.3% Triton X-100 in PBS for 30 min and blocked with 4% BSA in PBS for 30 min at room temperature. Sections were incubated overnight at 4 °C with anti-Iba1 antibody (1:500). After three washes in PBS for 10 min each, sections were incubated with Alexa Fluor 488 goat anti-rabbit IgG (1:500) for 2 h at room temperature in the dark. Nuclei were counterstained with DAPI for 10 min. Sections were mounted with 30% glycerol and imaged using a Zeiss LSM 900 confocal microscope with identical acquisition settings across all groups. Iba1-positive cells in the hippocampal DG region were counted using ImageJ. For each mouse, one hippocampal section was analyzed. Three mice per group were analyzed, and each mouse was treated as one biological replicate. Enlarged images of individual Iba1-positive microglia were used to visualize morphological alterations.

### Statistical analysis

Data are presented as mean ± SD. Sample sizes are indicated in the corresponding figure legends. Statistical analyses were performed using GraphPad Prism (version 10.1.2). For experiments with two independent variables (genotype and treatment), data were analyzed using two-way ANOVA followed by Tukey’s *post hoc* test. For comparisons involving a single experimental factor, one-way ANOVA with Tukey’s *post hoc* test was used. Statistical significance was defined as follows: ns, not significant (p > 0.05); *p < 0.05; **p < 0.01; ***p < 0.001.

### Data availability

All data supporting the study’s findings are available in the article and its supplementary materials.

## Results

### Exogenous L-fucose prevented CSDS-induced depressive-like behaviors in *Fut8^+/+^* mice

Previous studies have shown that Fut8 regulates neuroinflammatory signaling and stress-related depressive behaviors (32, 38), and that exogenous L-fucose suppresses microglial activation and inflammatory cytokine production in experimental models of neuroinflammation and CUS-induced depression (29, 39). However, whether L-fucose exerts protective effects under chronic social stress remains unclear. To address this question, we used the CSDS model, which recapitulates key behavioral and neuroinflammatory features associated with stress-related depression, including social avoidance, behavioral despair, and anhedonia (18, 19).

**Figure 1:**
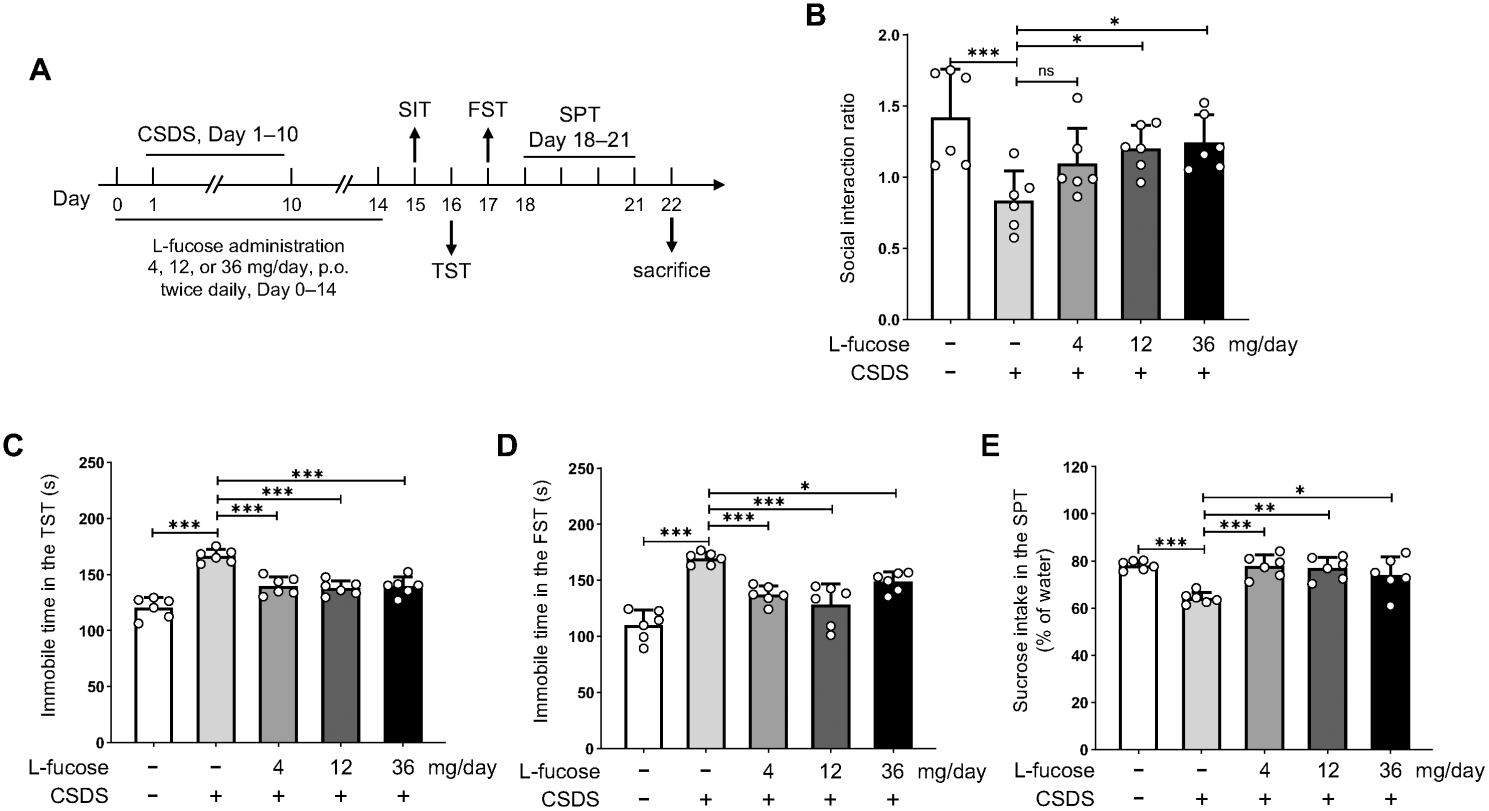
Exogenous L-fucose prevented CSDS-induced depressive-like behaviors in *Fut8^+/+^* mice. **(A)** Experimental timeline. Mice were subjected to CSDS and simultaneously administered L-fucose at the indicated doses (4, 12, or 36 mg/day) twice daily for 14 days. Behavioral tests were performed as described in the Methods, including the social interaction test (SIT), tail suspension test (TST), forced swimming test (FST), and sucrose preference test (SPT), followed by tissue collection. **(B)** Social interaction ratio in the SIT. **(C)** Immobility time in the TST. **(D)** Immobility time in the FST. **(E)** Sucrose preference in the SPT. Statistical analysis was performed using one-way ANOVA with Tukey’s multiple-comparison test. Data are presented as mean ± SD. n = 6 mice per group. ns, *p* > 0.05; *p < 0.05; **p < 0.01; ***p < 0.001.

To determine whether L-fucose exerts protective effects under chronic social stress, mice were subjected to CSDS while receiving L-fucose (preventive paradigm) at doses of 4, 12, or 36 mg/day for 14 consecutive days, followed by behavioral assessments (**Figure 1A**). CSDS exposure reduced social interaction and sucrose preference and increased immobility in both TST and FST, consistent with successful CSDS induction (**Figures 1B–E**). Notably, L-fucose administration ameliorated these behavioral deficits, as evidenced by increased SIT and SPT and reduced immobility in both behavioral despair paradigms. Given that microglial activation and pro-inflammatory cytokines are central to neuroinflammation-associated depression, we next examined inflammatory responses in brain tissues and found that CSDS significantly increased mRNA levels of *Il6*, *Il1β*, *Tnfα*, and *iNOS*. In comparison, L-fucose treatment suppressed these inflammatory changes (**Supplementary Figure 1**). Consistently, immunofluorescence analysis revealed that CSDS exposure increased the number of ionized calcium-binding adaptor molecule 1 (Iba1)–positive microglia and elevated *Iba1* mRNA expression. These alterations were attenuated by L-fucose administration (**Supplementary Figure 2**). Since the lowest dose (4 mg/day) already produced significant behavioral improvement, this dose was selected for subsequent experiments. These data support the conclusion that exogenous L-fucose prevents CSDS-induced depressive-like behaviors and suppresses neuroinflammatory responses accompanied by Iba1-positive microglial accumulation.

### Therapeutic L-fucose administration reversed established CSDS-induced depressive-like behaviors in *Fut8^+/+^* and *Fut8*^+/−^ mice

**Figure 2:**
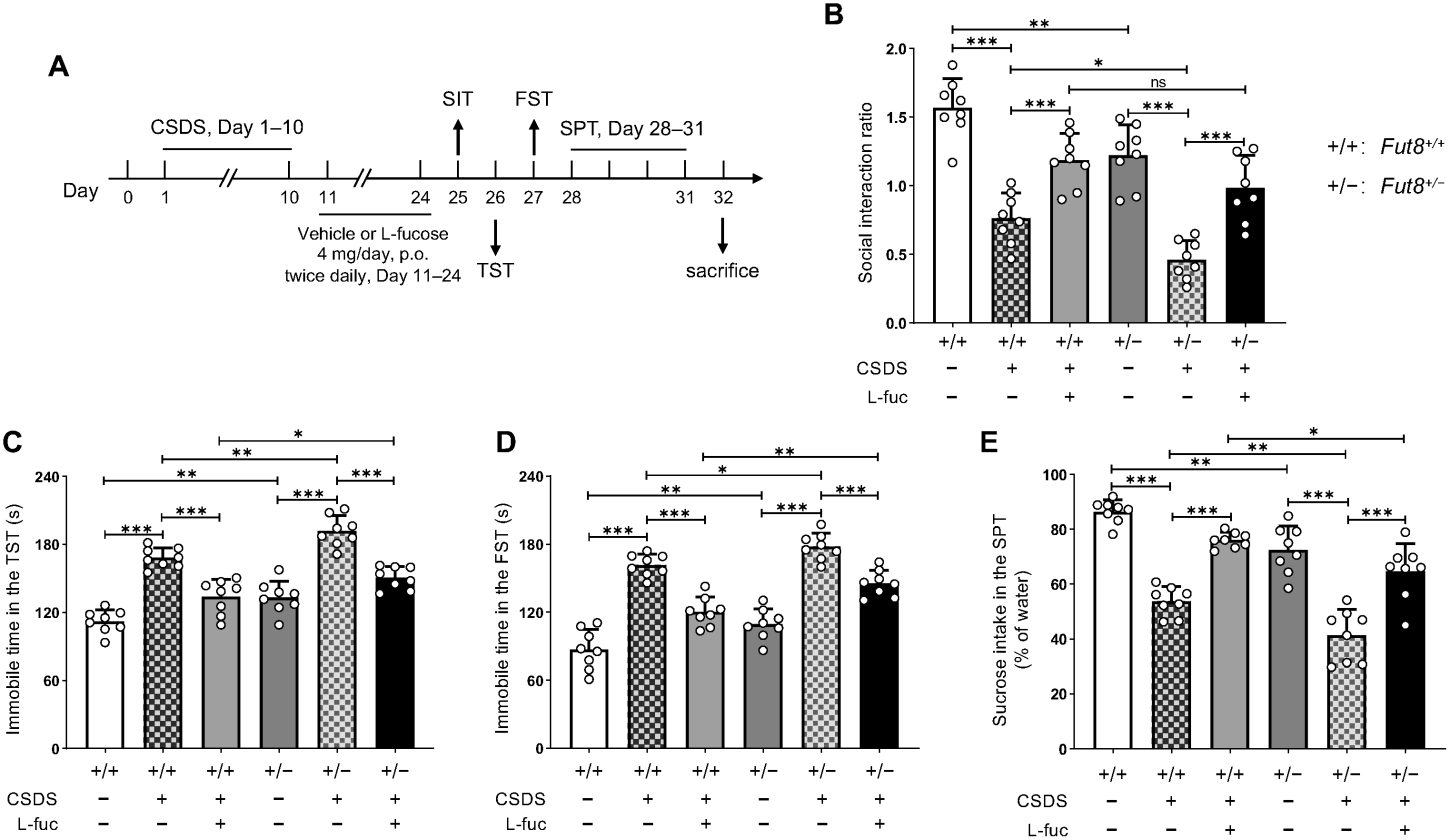
Therapeutic L-fucose administration reversed established CSDS-induced depressive-like behaviors in *Fut8^+/+^* and *Fut8^+/−^* mice. **(A)** Experimental design. *Fut8^+/+^* and *Fut8^+/−^* mice underwent CSDS for 10 consecutive days, followed by 2 weeks of oral L-fucose (4 mg/day). Behavioral tests were performed as described in the Methods. **(B)** Social interaction ratio in the SIT. **(C)** Immobility time in the TST. **(D)** Immobility time in the FST. **(E)** Sucrose preference in the SPT. All data are presented as mean ± SD and analyzed using two-way ANOVA with Tukey’s multiple-comparison test (n = 8 mice per group). ns, *p* > 0.05; *p < 0.05; **p < 0.01; ***p < 0.001.

To further evaluate whether L-fucose exerts therapeutic effects after stress exposure, we tested whether post-CSDS administration of L-fucose could ameliorate established behavioral abnormalities. In this paradigm, mice were first exposed to CSDS for 10 consecutive days (**Figure 2A**), followed by oral administration of L-fucose (4 mg/day) for an additional 14 days. To assess the contribution of core fucosylation, both *Fut8^+/+^* and *Fut8^+/−^* mice were included in the analysis. Mice exposed to CSDS exhibited reduced social interaction and sucrose preference, accompanied by increased immobility time in the TST and FST (**Figures 2B–E**). Subsequent L-fucose administration improved these behavioral abnormalities, as evidenced by increased social interaction ratios (**Figure 2B**) and sucrose preference (**Figure 2E**), together with reduced immobility time in both TST (**Figure 2C**) and FST (**Figure 2D**). Compared with *Fut8^+/+^* mice, *Fut8^+/−^* mice exhibited greater susceptibility to CSDS-induced behavioral impairments, including lower SIT and SPT, as well as prolonged immobility time. Although L-fucose administration improved these behavioral deficits in both genotypes, the magnitude of recovery appeared less pronounced in *Fut8^+/−^* mice than in *Fut8^+/+^* mice. These findings demonstrate that L-fucose administration exerted clear therapeutic effects on established CSDS-induced depressive-like phenotypes and further support a protective role of L-fucose supplementation against chronic stress-induced behavioral dysfunction.

### L-fucose attenuated CSDS-induced neuroinflammatory responses in the hippocampus of *Fut8^+/+^* and *Fut8^+/−^* mice

**Figure 3:**
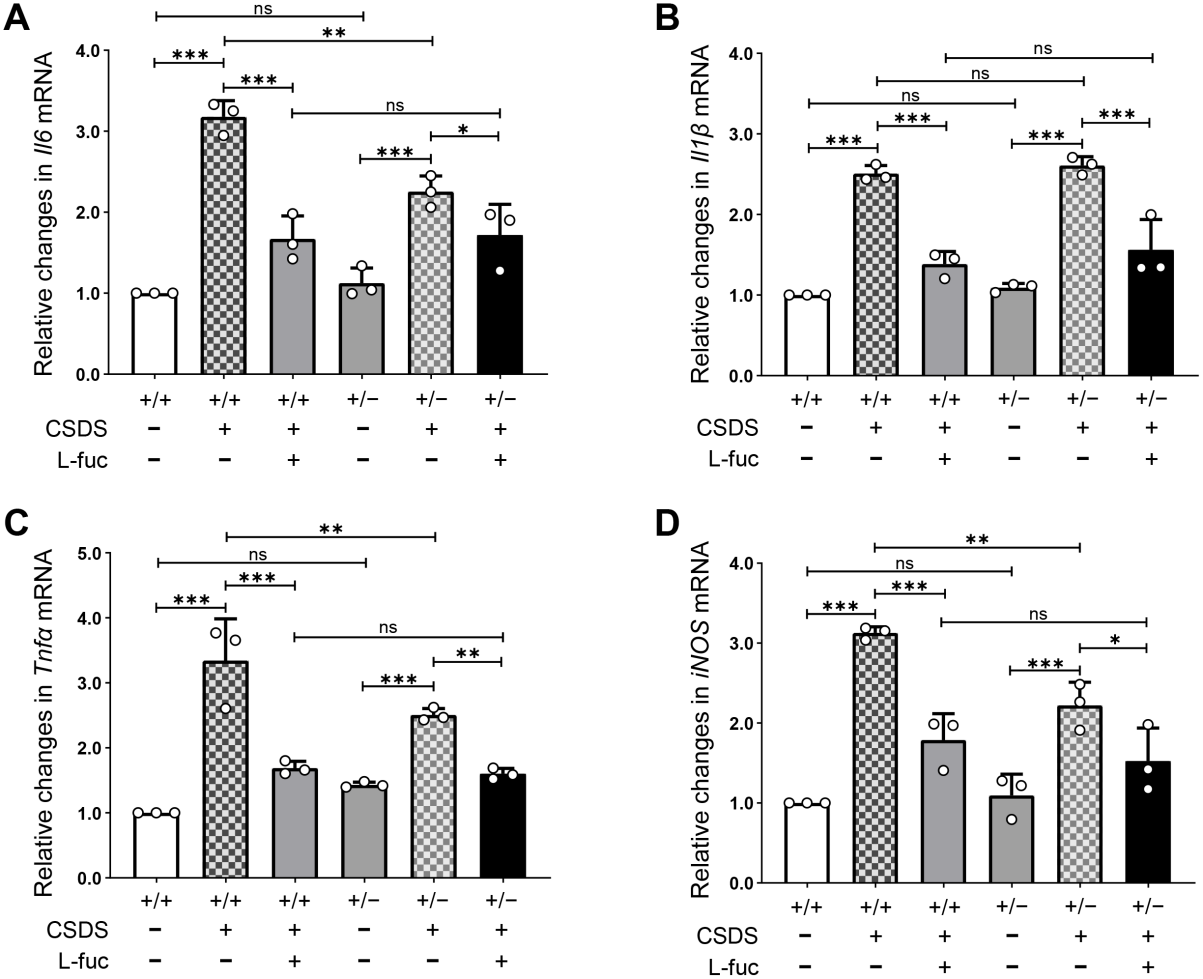
L-fucose attenuated CSDS-induced neuroinflammatory responses in the hippocampus of *Fut8^+/+^* and *Fut8^+/−^* mice. Pro-inflammatory cytokine mRNA levels were quantified by qPCR in hippocampal tissues from *Fut8^+/+^* and *Fut8^+/−^* mice subjected to CSDS with or without L-fucose administration. Relative expression changes in *Il6* **(A)**, *Il1β* **(B)**, *Tnfα* **(C)**, and *iNOS* **(D)** were measured and normalized to *Gapdh*. The ratio in *Fut8^+/+^* mice without CSDS and L-fucose administration was set to 1.0. All data are presented as mean ± SD (n = 3 mice per group). Statistical analysis was performed using two-way ANOVA with Tukey’s multiple-comparison test. ns, *p* > 0.05; *p < 0.05; **p < 0.01; ***p < 0.001.

Because chronic stress-induced behavioral abnormalities are closely associated with neuroinflammatory activation, we next analyzed inflammatory responses in the hippocampus. After CSDS, the mRNA expression of inflammatory cytokines increased in both *Fut8^+/+^* and *Fut8^+/−^* mice (**Figures 3A–D**). Compared with *Fut8^+/+^* mice, *Fut8^+/−^* mice exhibited a stronger inflammatory response after CSDS. Administration of L-fucose suppressed CSDS-induced upregulation of these inflammatory mediators in both genotypes (**Figures 3A–D**). These results suggest that exogenous L-fucose attenuates hippocampal inflammatory responses induced by chronic social stress.

### L-fucose suppressed JAK2/STAT3 signaling and microglial accumulation in the hippocampus of CSDS-exposed *Fut8^+/+^* and *Fut8^+/−^* mice

**Figure 4:**
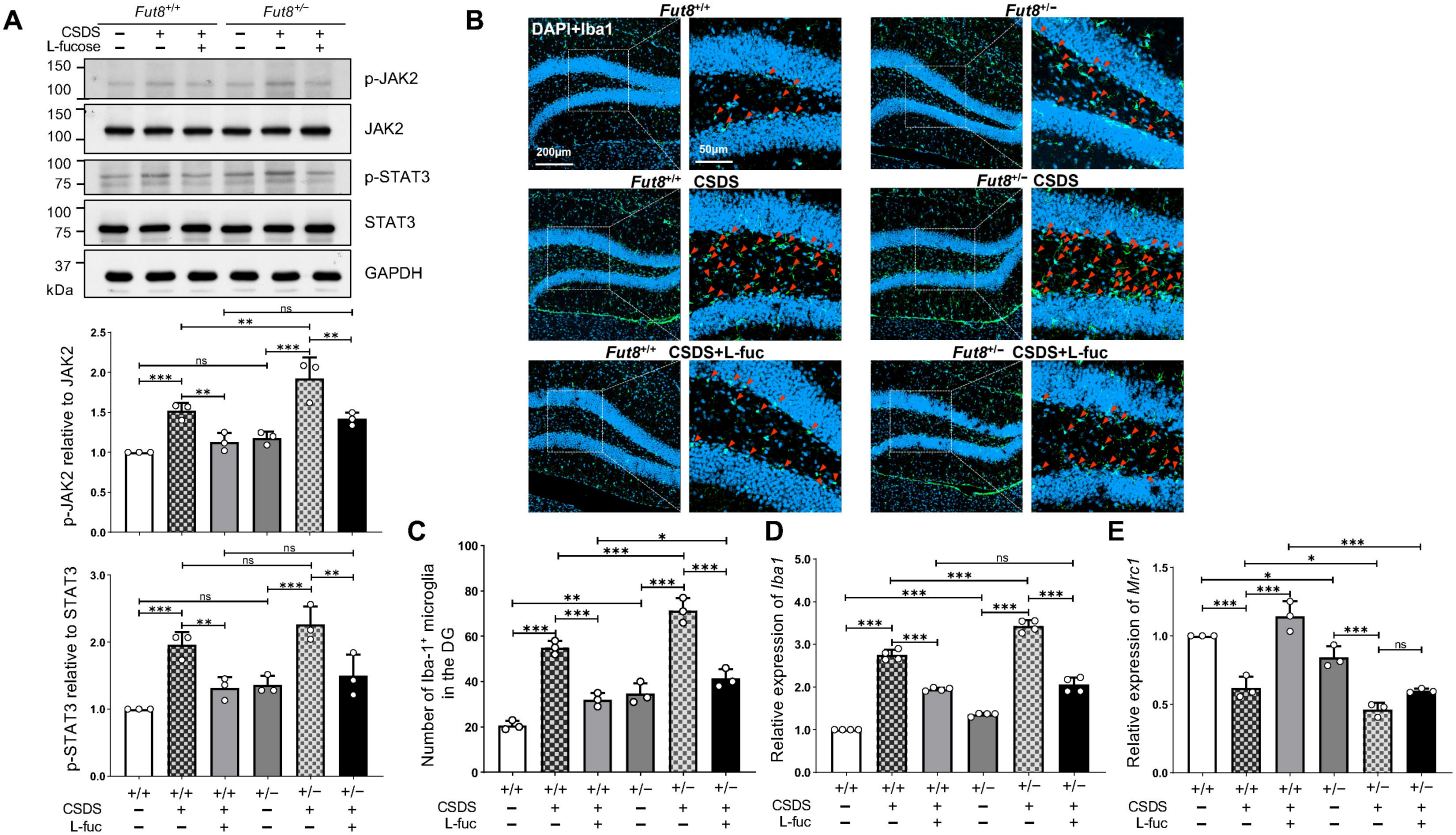
L-fucose suppressed JAK2/STAT3 signaling and microglial accumulation in the hippocampus of CSDS-exposed *Fut8^+/+^* and *Fut8^+/−^* mice. **(A)** Representative Western blot images of phosphorylated JAK2 (p-JAK2), total JAK2, phosphorylated STAT3 (p-STAT3), total STAT3, and GAPDH in hippocampal tissues from *Fut8^+/+^* and *Fut8^+/−^* mice with or without CSDS exposure and L-fucose administration. Quantitative data, measured with ImageJ, are shown below. **(B)** Representative immunofluorescence images of Iba1-positive microglia in the dentate gyrus (DG) of the hippocampus. White boxes and connecting lines indicate the regions shown at higher magnification. Red arrowheads indicate representative Iba1-positive microglia. Scale bars: 200 μm (left panels) and 50 μm (right panels). **(C)** Quantification of Iba1-positive microglia in the DG region using ImageJ. **(D)** qPCR analysis of *Iba1* mRNA levels in hippocampal tissues, normalized to *Gapdh* (internal control). *Fut8^+/+^* mice without CSDS exposure were set to 1.0. **(E)** Relative expression of *Mrc1/CD206* mRNA in hippocampal tissues. Statistical analyses were conducted using two-way ANOVA with Tukey’s multiple-comparison test. All data are presented as mean ± SD (n = 3 mice per group). ns, *p* > 0.05; *p < 0.05; **p < 0.01; ***p < 0.001.

Activation of the JAK2/STAT3 pathway has been closely linked to IL-6-mediated inflammatory signaling and microglial activation in the central nervous system (46, 47). Because IL-6 signaling is a major upstream activator of the JAK2/STAT3 pathway, we next examined whether CSDS affected JAK2/STAT3 signaling in the hippocampus of *Fut8^+/+^* and *Fut8^+/−^* mice. Western blot analysis showed that CSDS increased phosphorylation of JAK2 and STAT3, whereas total JAK2 and STAT3 protein levels remained largely unchanged (**Figure 4A**). The increases in phosphorylated JAK2 (p-JAK2) and phosphorylated STAT3 (p-STAT3) were more pronounced in *Fut8^+/−^* mice than in *Fut8^+/+^* mice, suggesting enhanced inflammatory signaling under conditions of reduced core fucosylation. Administration of L-fucose significantly suppressed CSDS-induced phosphorylation of both JAK2 and STAT3 (**Figure 4A**), indicating that L-fucose suppresses stress-induced JAK2/STAT3 activation.

Microglial activation is considered a key pathological feature of stress-related depressive disorders, and chronic stress has been reported to induce microglial alterations in specific hippocampal subregions (48, 49). Given the close relationship between STAT3 signaling and microglial inflammatory responses, we next evaluated Iba1-positive microglia in the hippocampal dentate gyrus (DG) using immunofluorescence staining (**Figure 4B**). Quantitative analysis showed that CSDS markedly increased the number of Iba1-positive microglia with a greater increase in *Fut8^+/−^* mice (**Figures 4B, C**). L-fucose administration reduced the number of Iba1-positive microglia after CSDS. Consistent with this, qPCR analysis showed that *Iba1* mRNA expression was elevated after CSDS and suppressed by L-fucose treatment (**Figure 4D**). To further assess inflammatory phenotypic changes, we examined the expression of *Mrc1*, which encodes mannose receptor C-type 1/CD206, an anti-inflammatory microglial/macrophage-associated marker. CSDS reduced *Mrc1* mRNA expression in the hippocampus, whereas L-fucose administration restored its expression in *Fut8^+/+^* mice and tended to partially increase it in *Fut8^+/−^* mice (**Figure 4E**). Together with the elevated hippocampal pro-inflammatory cytokine expression shown in **Figure 3**, these findings suggest that CSDS induces microglial accumulation accompanied by morphological alterations and inflammatory phenotypic changes. L-fucose suppresses CSDS-induced JAK2/STAT3 activation, reduces Iba1-positive microglial accumulation, and partially restores anti-inflammatory *Mrc1* expression, particularly under conditions of reduced core fucosylation.

### L-fucose restored hippocampal synaptic protein expression in *Fut8^+/+^* and *Fut8^+/−^* mice exposed to CSDS

**Figure 5:**
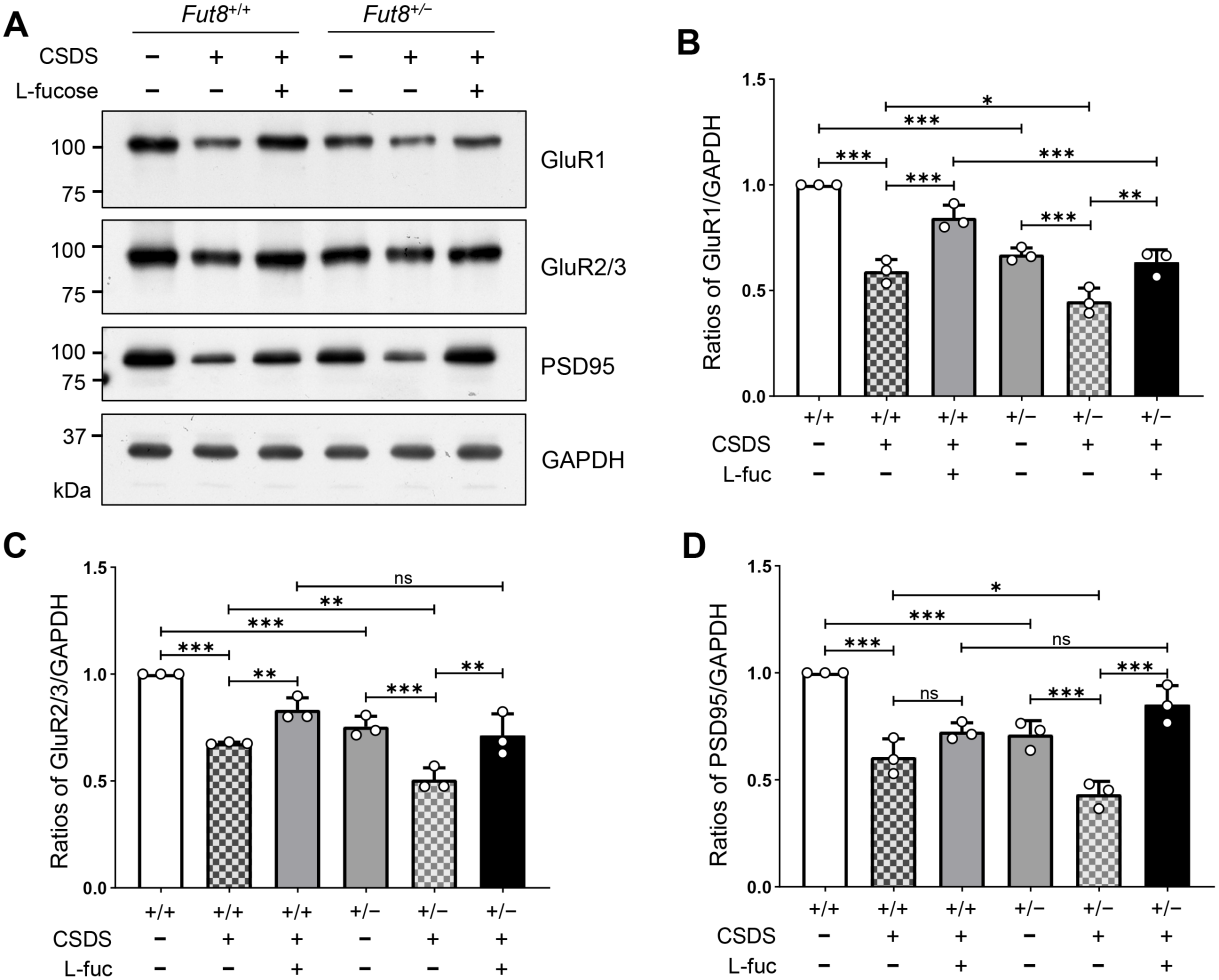
CSDS downregulated hippocampal synaptic protein expression, which was restored by L-fucose in *Fut8^+/+^* and *Fut8^+/−^* mice. **(A)** Western blot analysis of synaptic proteins in hippocampal tissues, including glutamate receptor 1 (GluR1), glutamate receptor 2/3 (GluR2/3), and postsynaptic density protein 95 (PSD95), quantified using ImageJ. **(B-D)** Quantification of protein expression levels normalized to glyceraldehyde-3-phosphate dehydrogenase (GAPDH), including GluR1 **(B)**, GluR2/3 **(C)**, and PSD95 **(D)**. GAPDH was used as a loading control, and the ratios of target proteins to GAPDH in *Fut8^+/+^* mice without CSDS exposure were set to 1.0. Statistical analyses were conducted using two-way ANOVA with Tukey’s multiple-comparison test. All data are presented as mean ± SD (n = 3 mice per group). ns, *p* > 0.05; *p < 0.05; **p < 0.01; ***p < 0.001.

To investigate whether L-fucose affects stress-induced synaptic alterations, we examined the expression of key excitatory synaptic proteins in the hippocampus after CSDS exposure. Western blot analysis showed reduced expression of the AMPA receptor subunits glutamate receptor 1 (GluR1; also termed GluA1) and glutamate receptor 2/3 (GluR2/3), as well as postsynaptic density protein 95 (PSD95), following CSDS exposure (**Figure 5A**). CSDS decreased hippocampal expression of GluR1, GluR2/3, and PSD95 in both *Fut8^+/+^* and *Fut8^+/−^* mice (**Figures 5B–D**), indicating impaired synaptic integrity under chronic stress. *Fut8^+/−^* mice showed more pronounced reductions in these proteins. Treatment with L-fucose partially restored the expression of these synaptic proteins in both genotypes after CSDS exposure (**Figures 5B–D**). These findings indicate that L-fucose attenuates CSDS-associated synaptic abnormalities in the hippocampus and may contribute to the improvement of depressive-like behaviors observed after CSDS exposure.

### Chronic stress reduced hippocampal GDP-fucose levels and impaired core fucosylation in *Fut8^+/+^* and *Fut8^+/−^* mice

**Figure 6:**
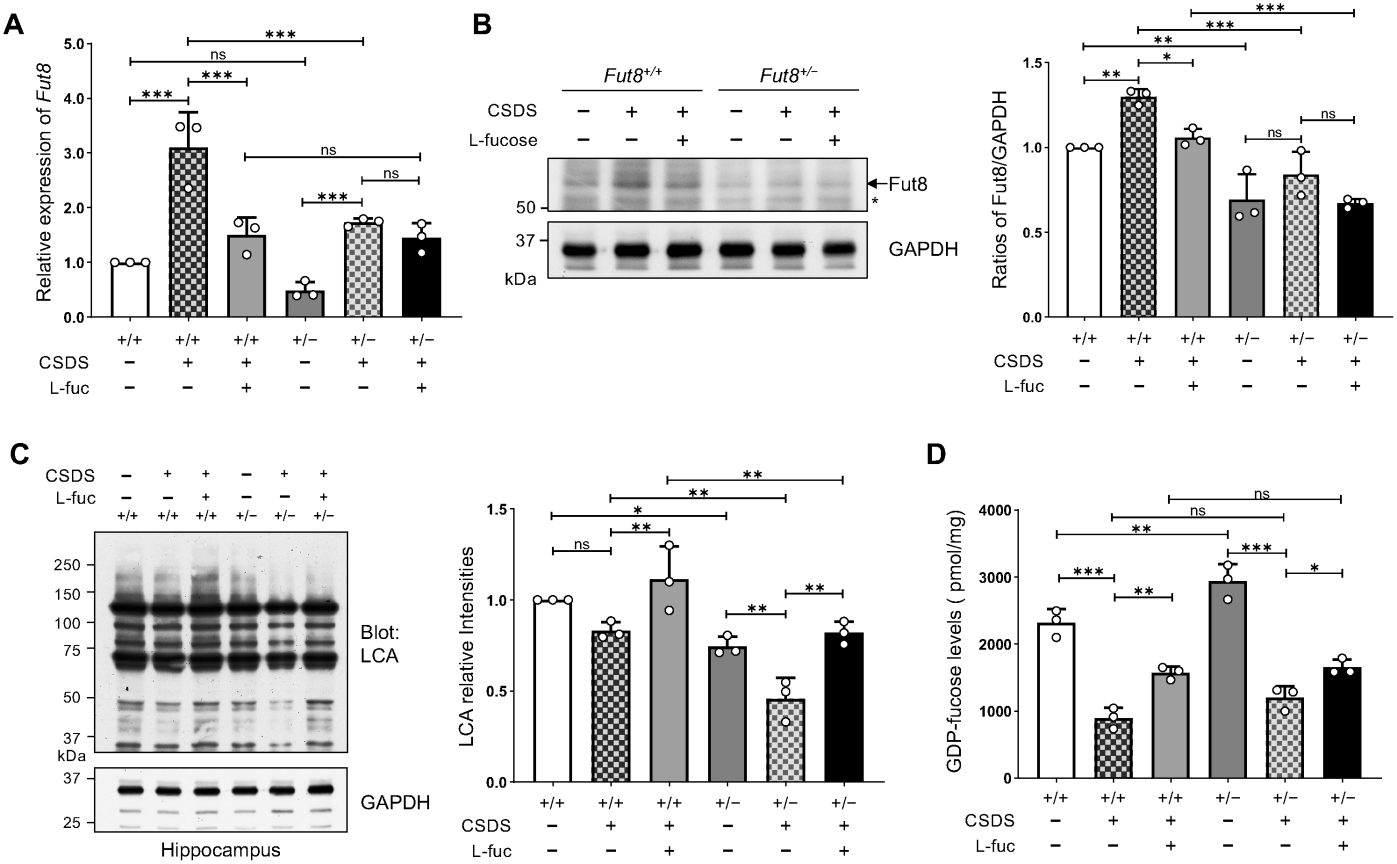
Chronic stress reduced hippocampal GDP-fucose levels and impaired core fucosylation in *Fut8^+/+^* and *Fut8^+/−^* mice. **(A)** *Fut8* mRNA levels were quantified by qPCR in hippocampal tissues from *Fut8^+/+^* and *Fut8^+/−^* mice subjected to CSDS with or without L-fucose administration. The ratio in *Fut8^+/+^* mice without CSDS and L-fucose administration was set to 1.0. **(B)** Representative Western blot images and quantification of Fut8 protein expression in hippocampal tissues. GAPDH served as a loading control. The arrow indicates the Fut8 band, and the asterisk indicates a nonspecific band. **(C)** Lectin blot analysis using Lens culinaris agglutinin (LCA) assessed core fucosylation levels in hippocampal tissues. A representative blot is shown in the left panel, and LCA signal intensities were quantified using ImageJ (right panel). GAPDH served as a loading control. The ratio in *Fut8^+/+^* mice without CSDS and L-fucose administration was set to 1.0. **(D)** GDP-fucose levels were quantified by HPLC in hippocampal tissues, as described in the Methods section. Data are presented as mean ± SD from three independent experiments. Group differences were evaluated using two-way ANOVA with Tukey’s multiple comparisons test (n = 3 mice per group). ns, *p* > 0.05; *p < 0.05; **p < 0.01; ***p < 0.001.

To determine whether chronic stress affects hippocampal core fucosylation, we first examined *Fut8* expression alongside LCA-reactive core fucosylation signals. qPCR analysis showed that CSDS increased *Fut8* mRNA expression in the hippocampus of *Fut8^+/+^* mice. In contrast, L-fucose administration partially suppressed this elevation (**Figure 6A**). As expected, basal *Fut8* expression was lower in *Fut8^+/−^* mice than in *Fut8^+/+^* mice, although CSDS also induced Fut8 upregulation in *Fut8^+/−^* mice. We next examined Fut8 protein expression by Western blotting. Consistent with the mRNA results, CSDS increased Fut8 protein levels in the hippocampus of *Fut8^+/+^* mice, and this increase was partially normalized by L-fucose administration (**Figure 6B**). In *Fut8^+/−^* mice, basal Fut8 protein levels were lower than in *Fut8^+/+^* mice, and CSDS did not induce a comparable increase in Fut8 protein expression. These results indicate that chronic stress alters Fut8 expression, particularly in *Fut8^+/+^* mice.

To further evaluate hippocampal core fucosylation status, lectin blot analysis using LCA was performed. Despite increased Fut8 expression after CSDS exposure, LCA-reactive signals were markedly reduced in the hippocampus, indicating impaired core fucosylation (**Figure 6C**). Administration of L-fucose restored LCA reactivity in *Fut8^+/+^* mice, whereas recovery was less evident in *Fut8^+/−^* mice. These findings indicate a dissociation between Fut8 expression and global core fucosylation under chronic stress conditions. Because core fucosylation depends on both Fut8 activity and intracellular GDP-fucose availability, hippocampal GDP-fucose levels were further analyzed by ion-pair reverse-phase high-performance liquid chromatography (HPLC). CSDS decreased hippocampal GDP-fucose levels, whereas L-fucose reversed this reduction in *Fut8^+/+^* mice (**Figure 6D**). A similar tendency was observed in *Fut8^+/−^* mice, although the restorative effect of L-fucose was less pronounced. To further examine whether chronic stress affected other fucosylated glycans, Lewis X-containing α1,3 fucosylation was analyzed by Western blotting with an anti-Lewis X antibody (**Supplementary Figure 3**). In contrast to the marked reduction in LCA reactivity, Lewis X showed only modest alterations across experimental groups, suggesting that distinct fucosylated glycans respond differently to chronic stress exposure (23, 50, 51). In addition, L-fucose administration produced only limited changes in Lewis X expression compared with the marked recovery observed in LCA reactivity, further supporting that Fut8 can preferentially utilize GDP-fucose derived from exogenous fucose, whereas GDP-fucose derived from endogenous fucose is used by other fucosyltransferases to modify *N*-glycan antennae (51). These findings suggest that impaired GDP-fucose availability, rather than reduced Fut8 expression, may contribute to chronic stress-associated disruption of core fucosylation.

### Chronic stress preferentially altered the GDP-fucose salvage pathway in the hippocampus

**Figure 7:**
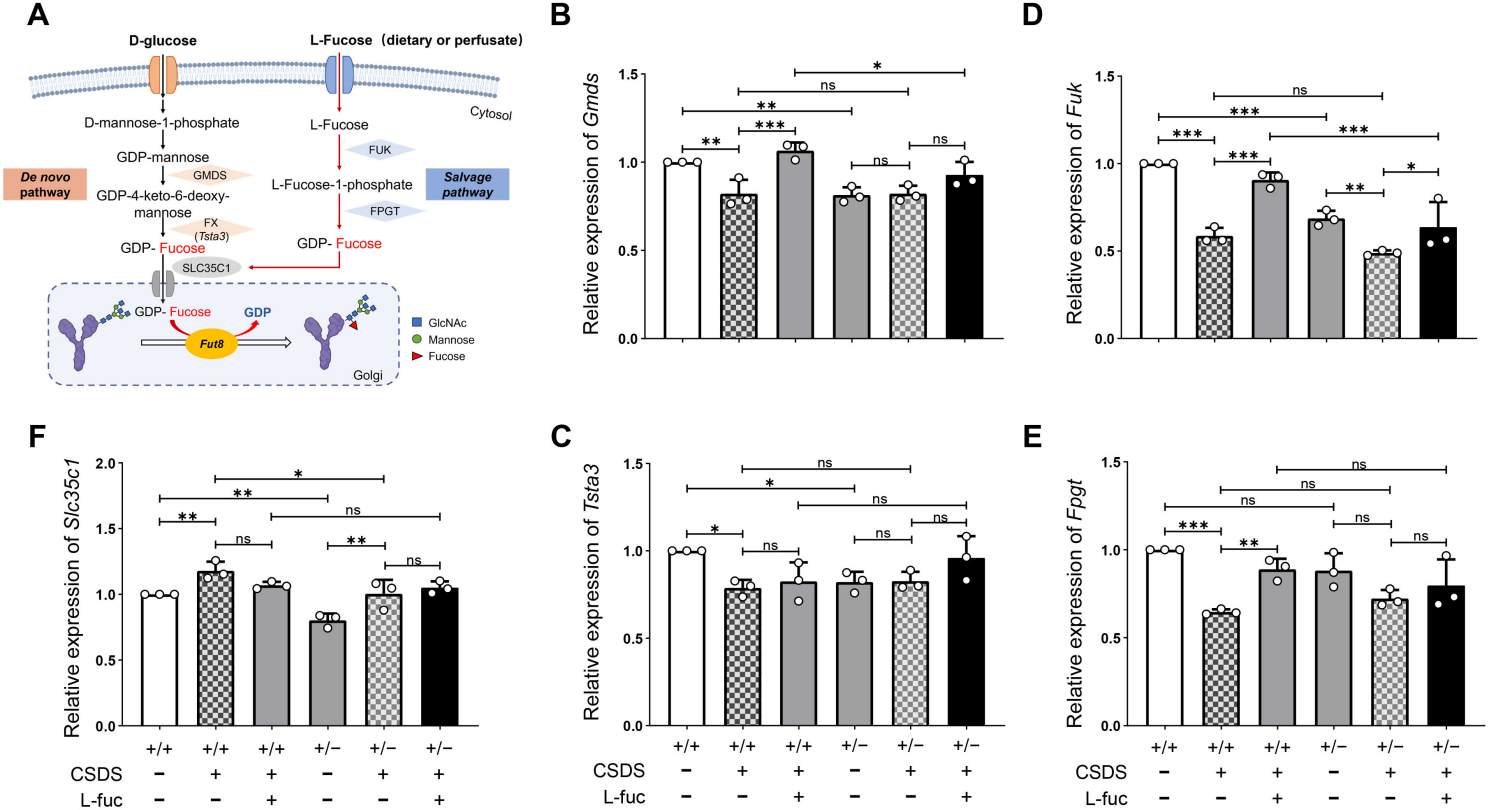
Chronic stress suppressed genes involved in GDP-fucose biosynthesis in the hippocampus, and this suppression was restored by L-fucose administration. **(A)** Schematic overview of intracellular GDP-fucose biosynthesis pathways and Fut8-mediated core fucosylation. GDP-fucose is generated through both the *de novo* and salvage pathways and transported into the Golgi via SLC35C1, where Fut8 catalyzes core fucosylation of glycoproteins. qPCR analysis of genes involved in GDP-fucose biosynthesis and transport, including GDP-mannose 4,6-dehydratase (*Gmds*) **(B)**, GDP-L-fucose synthase (*Tsta3)* **(C)**, Fucokinase (*Fuk*) **(D)**, Fucose-1-phosphate guanylyltransferase (*Fpgt*) **(E)**, and GDP-fucose transporter (*Slc35c1*) **(F)**, was performed, and mRNA levels were normalized to *Gapdh*. *Fut8^+/+^* mice without CSDS exposure were set to 1.0. Comparisons among groups were performed using two-way ANOVA followed by Tukey’s multiple-comparison test. All data are shown as mean ± SD from three independent experiments (n = 3 mice per group). ns, *p* > 0.05; *p < 0.05; **p < 0.01; ***p < 0.001.

To investigate the mechanism underlying stress-induced reductions in hippocampal GDP-fucose levels, we examined the expression of glycogenes involved in GDP-fucose biosynthesis and transport, including components of the *de novo* pathway (*Gmds* and *Tsta3*), the salvage pathway (*Fuk* and *Fpgt*), and the GDP-fucose transporter *Slc35c1* (31, 35) (**Figure 7A**). qPCR analysis revealed distinct changes in genes associated with GDP-fucose biosynthesis and transport following CSDS exposure (**Figures 7B–F**). In *Fut8^+/+^* mice, CSDS reduced the expression of *Fuk* and *Fpgt* (**Figures 7D, E**), whereas changes in *Gmds* expression were less pronounced (**Figure 7B**). In contrast, *Tsta3* expression remained largely unchanged (**Figure 7C**). Administration of L-fucose partially reversed CSDS-induced reductions in *Fuk* and *Fpgt* expression, whereas recovery of *Gmds* was modest. *Slc35c1* expression increased following CSDS exposure (**Figure 7F**). In *Fut8^+/−^* mice, the reduction in *Fuk* expression after CSDS exposure was more pronounced. However, alterations in *Gmds* and *Fpgt* expression were comparatively modest. L-fucose partially recovered the expression of *Fuk* and *Fpgt*, while *Slc35c1* expression remained elevated after both CSDS and L-fucose treatment. Similar to *Fut8^+/+^* mice, *Tsta3* expression showed minimal changes across experimental groups (**Figures 7B–F**). These transcriptional changes are consistent with the reduction in hippocampal GDP-fucose levels observed in **Figure 6D**. Overall, these findings suggest that chronic stress was associated with more pronounced alterations in the GDP-fucose salvage pathway; however, glycogenes associated with the *de novo* pathway were comparatively less sensitive to stress exposure. Although this interpretation remains speculative, the increase in *Slc35c1* expression may represent a compensatory response to reduced GDP-fucose availability (35, 52).

### Pharmacological inhibition of fucosylation with 2FF exacerbated stress-induced impairment of GDP-fucose availability and core fucosylation

**Figure 8:**
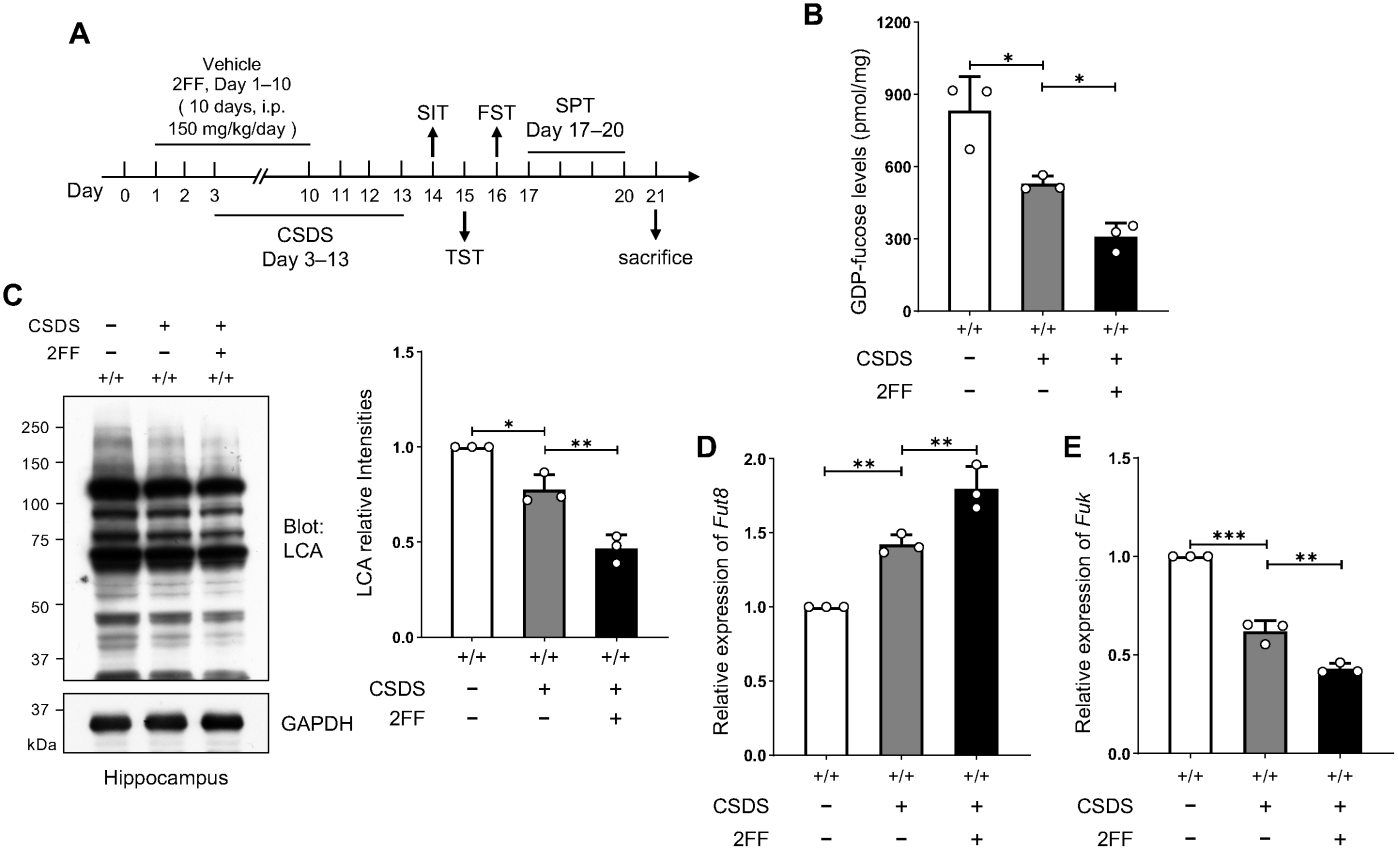
Pharmacological inhibition of fucosylation with 2FF exacerbated stress-induced impairment of GDP-fucose availability and core fucosylation. **(A)** Experimental timeline. *Fut8^+/+^* mice were subjected to CSDS and treated with 2-fluorofucose (2FF) or vehicle via intraperitoneal injection for 10 days. Behavioral tests were performed as indicated, and hippocampal tissues were collected for biochemical analyses. **(B)** Quantification of GDP-fucose levels in hippocampal tissues by HPLC. **(C)** Lectin blot analysis using LCA to assess core fucosylation levels in hippocampal tissues. LCA signal intensities were quantified with ImageJ and are shown in the right panel. GAPDH served as a loading control. qPCR analysis of *Fut8* **(D)** or *Fuk* **(E)** mRNA levels in hippocampal tissues, normalized to *Gapdh* (internal control). Relative expression in *Fut8^+/+^* mice without CSDS exposure was set to 1.0. All data are shown as mean ± SD from three independent experiments. Statistical analysis was performed using one-way ANOVA with Tukey’s multiple-comparison test (n = 3). ns, *p* > 0.05; *p < 0.05; **p < 0.01; ***p < 0.001.

We next examined whether pharmacological inhibition of fucosylation further exacerbated the stress-associated reduction in hippocampal GDP-fucose and core fucosylation using 2-fluorofucose (2FF), a fucose analog that inhibits cellular fucosylation through metabolic conversion to GDP-2FF (53) (**Figure 8A**). HPLC analysis showed that CSDS lowered hippocampal GDP-fucose levels, consistent with the changes observed in **Figure 6**. Treatment with 2FF further decreased hippocampal GDP-fucose levels beyond those induced by CSDS alone (**Figure 8B**), indicating an additional reduction in GDP-fucose availability following inhibition of cellular fucosylation. LCA lectin blot analysis further demonstrated that CSDS reduced hippocampal LCA reactivity, whereas 2FF treatment induced a more pronounced reduction in LCA-reactive signals (**Figure 8C**). These results suggest that pharmacological inhibition of fucosylation further exacerbates stress-associated impairment of core fucosylation. We also examined the expression of glycogenes associated with GDP-fucose synthesis and core fucosylation. Consistent with the findings in **Figure 6**, *Fut8* expression increased after CSDS exposure and was further elevated following 2FF administration, despite progressive reductions in core fucosylation (**Figure 8D**). In contrast, *Fuk* expression was reduced after CSDS exposure and was further suppressed by 2FF administration (**Figure 8E**). Taken together, these findings demonstrate that pharmacological inhibition of fucosylation exacerbates chronic stress-induced reductions in hippocampal GDP-fucose levels and core fucosylation and is accompanied by further suppression of Fuk expression.

### 2FF exacerbated CSDS-induced behavioral abnormalities and hippocampal inflammation in *Fut8^+/+^* mice

**Figure 9:**
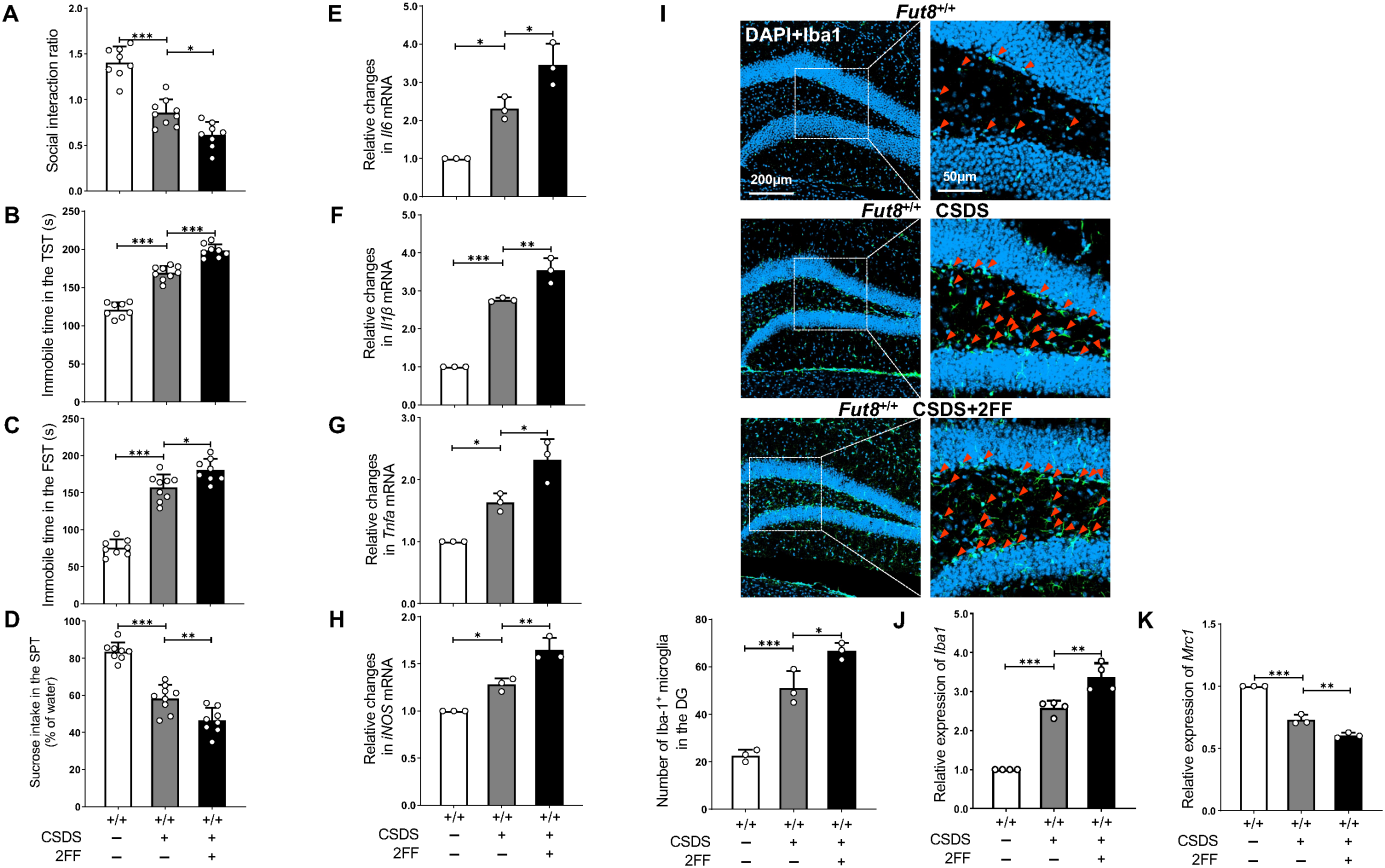
2FF exacerbated CSDS-induced behavioral abnormalities and hippocampal inflammation in *Fut8^+/+^* mice. Behavioral tests were conducted in *Fut8^+/+^* mice subjected to CSDS with or without 2FF. **(A)** Social interaction ratio in the SIT. **(B)** Immobility time in the TST. **(C)** Immobility time in the FST. **(D)** Sucrose preference in the SPT. Pro-inflammatory cytokine mRNA levels were quantified by qPCR in hippocampal tissues, including *Il6* **(E)**, *Il1β* **(F)**, *Tnfα* **(G)**, and *iNOS* **(H)**. mRNA levels were normalized to *Gapdh*, and *Fut8^+/+^* mice without CSDS exposure were set to 1.0. **(I)** Representative immunofluorescence images of Iba1 in the DG of the hippocampus. Iba1 was used as a microglial marker, and DAPI was used for nuclear staining. White boxes and connecting lines indicate the regions shown at higher magnification. Red arrowheads indicate representative Iba1-positive microglia. Scale bars: 200 μm (left panels) and 50 μm (right panels). Quantification of Iba1-positive microglia in the DG region is shown below. **(J)** Relative expression of *Iba1* mRNA was quantified by qPCR. **(K)** Relative expression of *Mrc1* mRNA in hippocampal tissues. All data are presented as mean ± SD, with statistical evaluation performed by one-way ANOVA with Tukey’s multiple-comparison test. For behavioral analyses, n = 8 mice per group; for qPCR and histological analyses, n = 3 mice per group. ns, *p* > 0.05; *p < 0.05; **p < 0.01; ***p < 0.001.

We next examined whether 2FF treatment affected behavioral and inflammatory alterations induced by CSDS. Behavioral testing revealed that CSDS decreased social interaction and sucrose preference and increased immobility time in both the TST and FST (**Figures 9A–D**). Treatment with 2FF further worsened these behavioral deficits. Hippocampal inflammatory responses were then analyzed by qPCR. Although exposure to CSDS significantly elevated hippocampal expression of *Il6*, *Il1β*, *Tnfα*, and *iNOS* mRNAs, 2FF treatment further increased the expression of these inflammatory mediators (**Figures 9E–H**), indicating enhanced neuroinflammatory responses following pharmacological inhibition of fucosylation. Immunofluorescence staining showed that the number of Iba1-positive microglia in the DG increased after CSDS exposure and was further elevated by 2FF treatment (**Figure 9I**). Quantitative analysis further confirmed an increase in Iba1-positive microglia and elevated *Iba1* mRNA expression after 2FF administration in CSDS-exposed mice (**Figure 9J**). To further assess inflammatory phenotypic changes, we examined *Mrc1* expression. CSDS reduced *Mrc1* mRNA expression in the hippocampus, and 2FF treatment further suppressed *Mrc1* expression under CSDS conditions (**Figure 9K**). These findings suggest that impaired fucosylation exacerbates stress-associated behavioral abnormalities, hippocampal inflammatory responses, and microglial accumulation accompanied by inflammatory phenotypic changes. However, because 2FF is not Fut8-specific, these results do not establish that the aggravated phenotypes are exclusively mediated by selective inhibition of Fut8-dependent core fucosylation.

### L-fucose or MF30 attenuated CSDS-induced inflammatory responses, and MF30 improved depressive-like behaviors in mice exposed to CSDS

**Figure 10:**
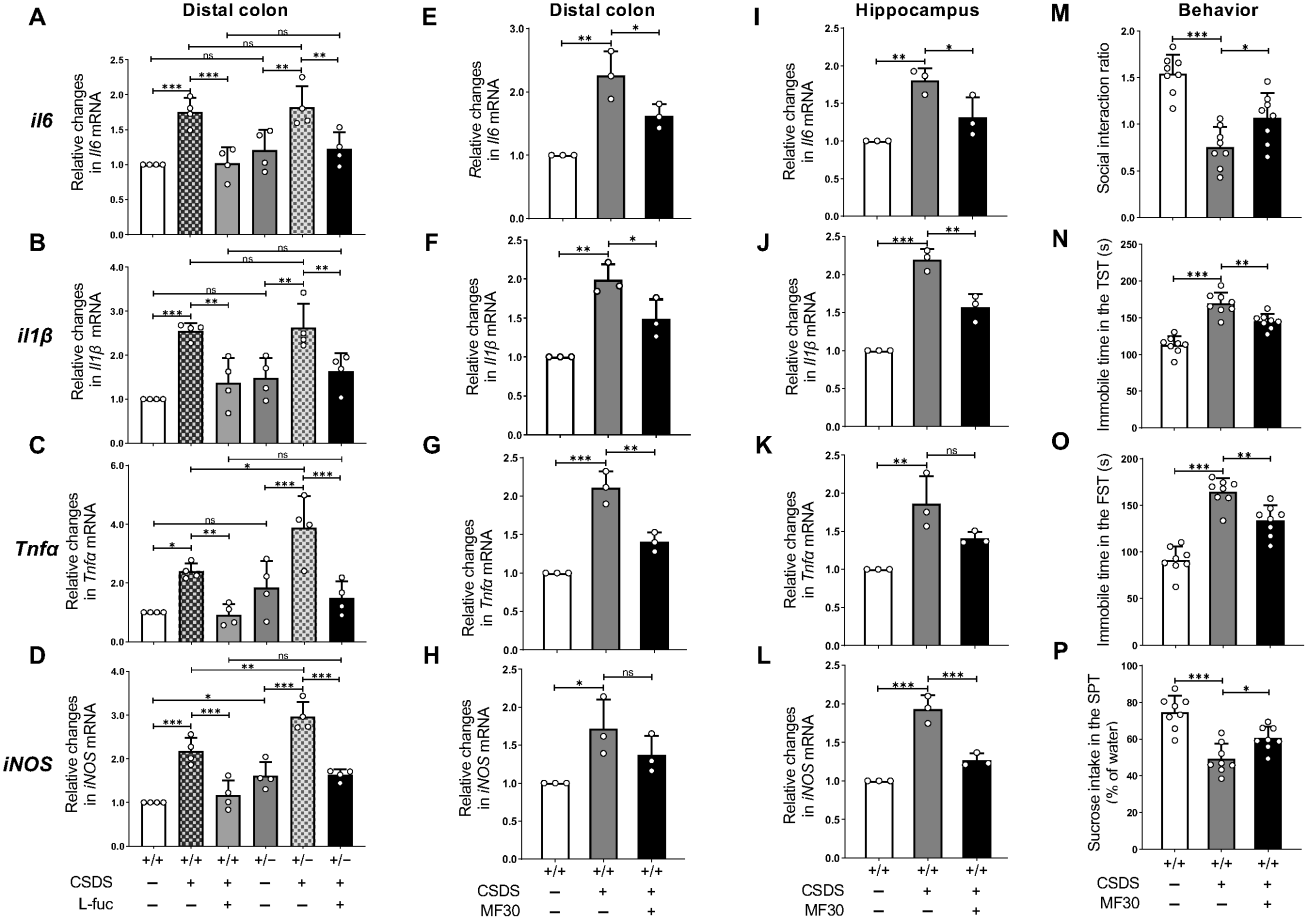
L-fucose or MF30 administration attenuated CSDS-induced inflammatory responses, and MF30 improved depressive-like behaviors in CSDS-exposed mice. Pro-inflammatory cytokine mRNA levels were quantified by qPCR. **(A–D)** *Il6* **(A)**, *Il1β* **(B)**, *Tnfα* **(C)**, and *iNOS* **(D)** mRNA levels in distal colon tissues from *Fut8^+/+^* and *Fut8^+/−^* mice subjected to CSDS with or without L-fucose administration. **(E–H)** *Il6* **(E)**, *Il1β* **(F)**, *Tnfα* **(G)**, and *iNOS* **(H)** mRNA levels in distal colon tissues from *Fut8^+/+^* mice subjected to CSDS with or without MF30 administration. **(I–L)** *Il6* **(I)**, *Il1*β **(J)**, *Tnf*α **(K)**, and *iNOS* **(L)** mRNA levels in hippocampal tissues from *Fut8^+/+^* mice subjected to CSDS with or without MF30 administration. **(M–P)** Behavioral analyses of CSDS-exposed *Fut8^+/+^* mice treated with or without MF30, including social interaction ratio **(M)**, immobility time in the TST **(N)**, immobility time in the FST **(O)**, and sucrose preference in the SPT **(P)**. Relative mRNA levels were normalized to *Gapdh*, and *Fut8^+/+^* mice without CSDS exposure were set to 1.0. All data are expressed as mean ± SD. For qPCR analyses, n = 3 or 4 mice per group; for behavioral analyses, n = 8 mice per group. Statistical comparisons for panels A–D were performed using two-way ANOVA with Tukey’s multiple-comparison test, whereas panels E–P were performed using one-way ANOVA with Tukey’s multiple-comparison test. ns, *p* > 0.05; *p < 0.05; **p < 0.01; ***p < 0.001.

Given the close relationship between chronic stress and systemic inflammation (54), we examined whether CSDS also induced inflammatory responses in the distal colon. The distal colon was selected because it is highly enriched in immune cells and is sensitive to stress-associated inflammatory changes linked to gut–brain axis signaling (28, 55). qPCR analysis showed that CSDS increased mRNA expression of *Il6*, *Il1β*, *Tnfα*, and *iNOS* in the distal colon (**Figures 10A–H**). *Fut8^+/−^* mice exhibited more pronounced inflammatory responses after CSDS exposure. Exogenous L-fucose or MF30, a seaweed-derived fucose-containing dietary preparation, significantly suppressed CSDS-induced upregulation of these inflammatory mediators in both *Fut8^+/+^* and *Fut8^+/−^* mice (**Figures 10A–H**). Like L-fucose, MF30 also suppressed these inflammatory responses in the hippocampal tissues (**Figures 10I–L**). To further determine whether MF30 exerts protective effects against CSDS-induced behavioral abnormalities, we performed behavioral analyses in CSDS-exposed mice treated with MF30. MF30 administration significantly increased the social interaction ratio and sucrose preference, while reducing immobility time in the TST and FST (**Figures 10M–P**). These results demonstrate that chronic stress induced inflammatory responses not only in the hippocampus but also in peripheral tissues, whereas L-fucose or MF30 treatment attenuated both central and peripheral inflammatory alterations. However, whether these central and peripheral inflammatory changes are causally linked remains to be investigated.

## Discussion

**Figure 11:**
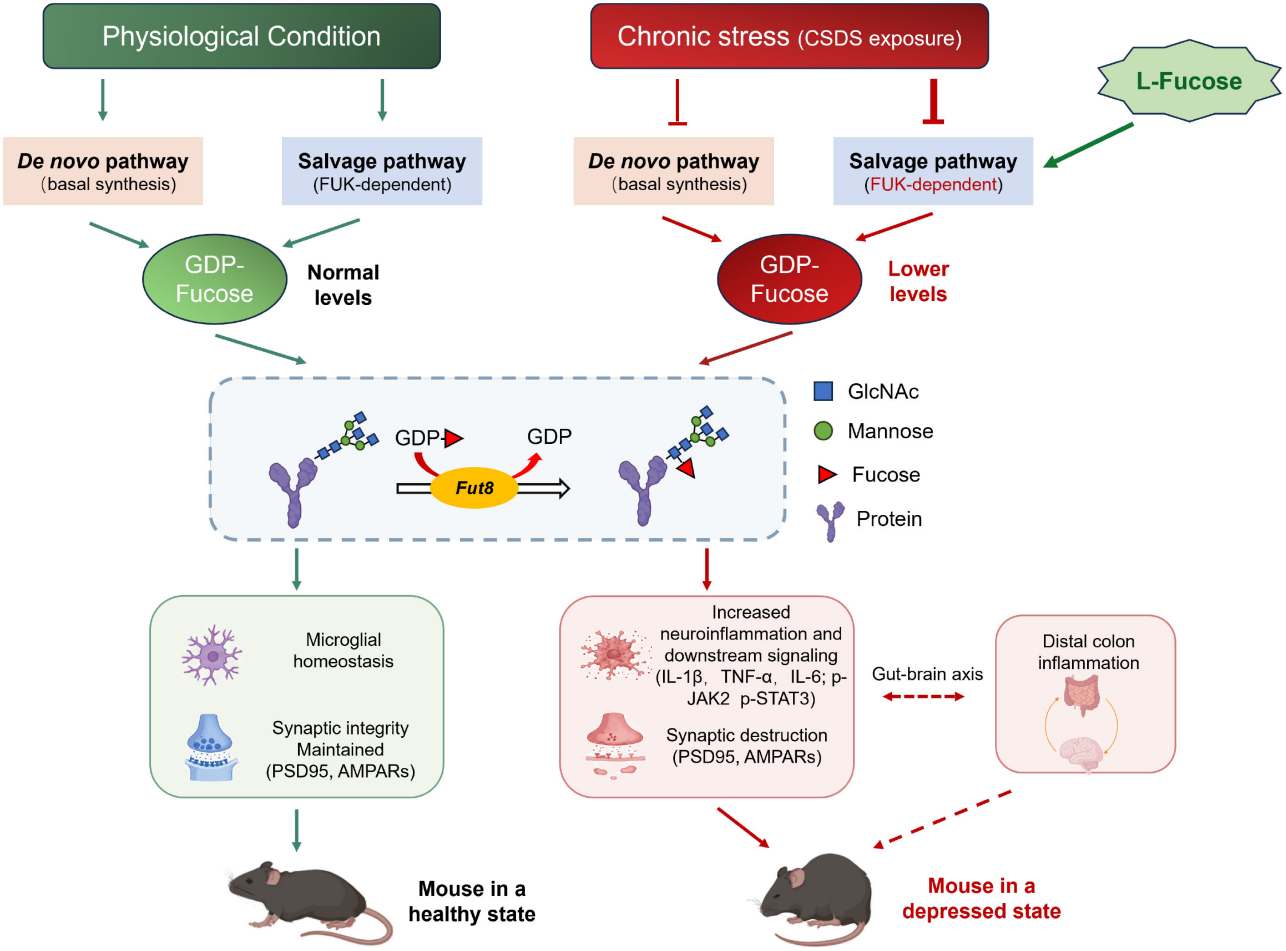
Proposed model illustrating chronic stress-induced depressive-like behaviors, in which regulation of GDP-fucose biosynthesis and core fucosylation plays crucial roles. Under physiological conditions, both the *de novo* and salvage pathways help maintain intracellular GDP-fucose levels, supporting Fut8-mediated core fucosylation and normal neuroimmune and synaptic homeostasis. Following CSDS, salvage pathway-associated biosynthesis is preferentially impaired, leading to depletion of hippocampal GDP-fucose and reduced core fucosylation. Impaired core fucosylation is associated with enhanced neuroinflammatory responses, increased JAK2/STAT3 signaling, microglial accumulation, synaptic dysfunction, and depressive-like behaviors. Exogenous L-fucose significantly restores GDP-fucose levels and core fucosylation, alleviating stress-associated neuroinflammatory and behavioral abnormalities. Furthermore, CSDS induces inflammatory responses in distal colon tissues, and this inflammation can be suppressed not only by L-fucose but also by L-fucose-containing dietary preparations, such as MF30, underscoring the importance of the gut-brain axis. Dashed lines indicate uncertainty in this study so far, and further study is required. This figure was created with BioRender.com.

Major depressive disorder is increasingly recognized as a stress-associated neuroinflammatory disorder, yet the metabolic mechanisms linking chronic psychosocial stress to sustained CNS immune dysregulation remain incompletely understood (49, 56). In the present study, our findings provide new evidence that reduced hippocampal GDP-fucose biosynthesis and core fucosylation are associated with CSDS-induced depressive-like behaviors. CSDS reduced hippocampal GDP-fucose levels by downregulating the salvage pathway-associated enzymes Fuk and Fpgt, thereby decreasing core fucosylation and resulting in enhanced neuroinflammatory responses, synaptic abnormalities, and depressive-like behaviors (**Figure 11**). These effects were significantly rescued by L-fucose supplementation (**Figure 11**). These findings extend previous observations from LPS- and CUS-induced models of CNS dysfunction to a socially relevant chronic stress setting (29, 39). Rather than identifying GDP-fucose metabolism as an isolated or exclusive mechanism, the present results support a model in which the FUK/FPGT-dependent salvage pathway may act as a stress-responsive glycometabolic component that interacts with established neuroinflammatory and synaptic pathways during chronic stress.

Notably, in this study, CSDS decreased core fucosylation despite increased Fut8 expression (**Figures 6A, B**). This apparent discrepancy suggests that Fut8 expression alone is insufficient to maintain core fucosylation under chronic stress. In fact, *Fut8* mRNA expression has been reported to be upregulated during the acute phase but downregulated during the late phase, as observed in the CUS and BV2 cell models (39). Considering that Fut8-mediated core fucosylation depends not only on Fut8 expression but also on intracellular GDP-fucose availability, reduced GDP-fucose levels may become a limiting factor during chronic stress exposure. To further examine this possibility, HPLC analysis showed that CSDS markedly reduced hippocampal GDP-fucose levels, whereas L-fucose supplementation partially recovered both GDP-fucose abundance and core fucosylation (**Figure 6**). In addition, unlike core fucosylation, the expression of Lewis X-containing α1,3 fucosylation exhibited only modest alterations after CSDS exposure (**Supplementary Figure 3**) or L-fucose administration, suggesting that chronic stress does not uniformly affect all fucosylated glycans, and that core fucosylation appears particularly sensitive to alterations in GDP-fucose metabolism (51). These observations indicate that intracellular GDP-fucose availability, rather than Fut8 expression alone, may become rate-limiting for core fucosylation during chronic stress.

Compared with enzymes associated with the *de novo* pathway, those associated with the salvage pathway appeared more sensitive to chronic stress exposure (**Figure 7**). In particular, CSDS reduced expression of the salvage pathway-associated enzymes Fuk and Fpgt; however, enzymes involved in the *de novo* pathway remained relatively stable, suggesting differential regulation of GDP-fucose biosynthetic pathways under chronic stress conditions (31, 35, 52). Because the salvage pathway uses free L-fucose to replenish intracellular GDP-fucose pools, reduced salvage pathway activity may limit the capacity to maintain GDP-fucose availability under stress-related metabolic demand. The increase in *Slc35c1* expression may instead reflect a compensatory response to declining intracellular GDP-fucose availability, although this response alone appeared insufficient to preserve core fucosylation. Interestingly, the relatively higher GDP-fucose levels observed in *Fut8^+/−^* mice may partly reflect reduced Fut8-mediated GDP-fucose utilization. It should be noted that 2FF is not a Fut8-specific inhibitor, since it can be metabolized to GDP-2FF and broadly inhibits cellular fucosylation. The 2FF results should be interpreted as complementary pharmacological evidence that global suppression of fucosylation exacerbates CSDS-induced glycometabolic, behavioral, and inflammatory abnormalities, rather than as evidence for a Fut8-specific mechanism. Together with the *Fut8^+/−^* mouse data and LCA-based analysis, these findings support the possibility that impaired fucosylation, including reduced core fucosylation, is associated with increased vulnerability under chronic stress conditions. Collectively, these observations support an association among chronic psychosocial stress, suppression of the FUK/FPGT-dependent salvage pathway, reduced GDP-fucose availability, and impaired core fucosylation. The mechanisms by which chronic psychosocial stress suppresses FUK and FPGT expression require further investigation.

Chronic stress is known to activate multiple converging pathways, including HPA-axis dysregulation, glucocorticoid signaling abnormalities, microglial priming, and sustained cytokine production (57–59). In this study, impaired GDP-fucose metabolism is unlikely to constitute an entirely separate pathway. Instead, reduced GDP-fucose availability and impaired core fucosylation may complement or amplify these established stress-responsive mechanisms by modulating glycosylation-dependent receptor signaling, cytokine responsiveness, and microglial inflammatory activation. CSDS increased hippocampal inflammatory cytokine expression and Iba1-positive microglial accumulation in the DG (**Supplementary Figures 1, 2**). These inflammatory changes were more pronounced in *Fut8^+/−^* mice (**Figures 3**, **4B**), suggesting that reduced core fucosylation increases vulnerability to chronic stress-associated neuroinflammatory responses. However, L-fucose supplementation attenuated cytokine expression and Iba1-positive microglial accumulation, supporting the idea that restoring fucose metabolism can restrain stress-induced CNS inflammation. These results are consistent with previous reports showing that L-fucose and core fucosylation exert anti-inflammatory effects in models of neuroinflammation and depression (29, 39). Furthermore, dysregulation of core fucosylation has also been reported in several neuroinflammatory and neurodegenerative disorders, including Alzheimer’s disease, schizophrenia, and multiple sclerosis (50, 60, 61), supporting a broader role for altered glycosylation in CNS inflammatory dysfunction (23).

Mechanistically, altered core fucosylation may influence inflammatory signaling through receptor-dependent pathways. Previous studies have suggested that impaired core fucosylation can enhance IL-6R/gp130-associated inflammatory signaling (29, 37, 62). Similar trends were observed in the present study, in which CSDS enhanced JAK2/STAT3 signaling in the hippocampus, as evidenced by increased phosphorylation of both proteins, whereas L-fucose administration partially reversed these changes (**Figure 4A**). In contrast, pharmacological inhibition of fucosylation with 2FF further aggravated neuroinflammation and depressive-like behaviors in CSDS-exposed mice (**Figures 8**, **9**). Although multiple cell-surface glycoproteins are presumably affected during chronic stress exposure, the present findings, together with our previous work showing that Fut8-mediated core fucosylation regulates IL-6R/gp130, support the possibility that dysregulated inflammatory signaling is a downstream consequence of impaired core fucosylation. To further understand the underlying molecular mechanisms, a comprehensive glycoproteomic analysis is required in the future.

Neuroinflammation is closely linked to synaptic dysfunction in depression (63, 64). Activated microglia release inflammatory mediators that can disrupt excitatory neurotransmission and synaptic stability. In the present study, CSDS reduced the expression of synaptic proteins, including GluR1, GluR2/3, and PSD95. In contrast, L-fucose supplementation partially reversed these changes (**Figure 5**). A recent study also showed that L-fucose may function as a neuromodulatory molecule in the CNS (30). These findings are consistent with stress-associated synaptic impairment and suggest that altered fucose metabolism may contribute to disruption of excitatory synaptic homeostasis. Previous studies have shown that impaired core fucosylation disrupts AMPA receptor-associated signaling and hippocampal synaptic plasticity, supporting the possibility that altered receptor glycosylation contributes to stress-associated synaptic dysfunction (22, 65–67). Together, these observations support a plausible synergistic model in which persistent neuroinflammatory activation and impaired glycosylation-dependent receptor regulation may jointly contribute to changes in synaptic proteins during chronic stress. The detailed mechanism requires further study.

In this study, the distal colon was selected as a peripheral immune-related tissue because it is highly enriched in immune cells and closely associated with microbiota-dependent immune regulation (68). In addition to central neuroinflammatory alterations, CSDS increased pro-inflammatory cytokine expression in the distal colon. These peripheral immune alterations were more pronounced in *Fut8^+/−^* mice and were attenuated by supplementation with L-fucose or MF30, a marine-derived fucose-enriched seaweed hydrolysate (**Figure 10**). These findings indicate that CSDS is associated with inflammatory alterations not only in the hippocampus but also in peripheral intestinal tissue. Because MF30 contains dextrin and other seaweed-derived carbohydrate components in addition to L-fucose, potential effects on gut microbial metabolism cannot be excluded. Certain dextrin-containing dietary fibers have been reported to modulate gut microbial composition and microbial metabolite production, although whether these components contribute to the protective effects of MF30 remains unclear (69). These findings suggest that fucose metabolism may also influence stress-associated peripheral immune dysregulation. Because intestinal immune homeostasis is strongly influenced by glycan-mediated host–microbe interactions, altered fucosylation-related pathways may affect gut immune responses during chronic stress exposure (70, 71). Intestinal microbes extensively utilize fucose-containing glycans as metabolic substrates, and altered fucose metabolism may influence host–microbe interactions under stress conditions (28, 72). Increasing evidence suggests that chronic stress-associated intestinal inflammation and microbiota dysregulation can influence CNS immune responses through gut–brain axis-associated signaling pathways (73, 74). Although microbiota composition and gut permeability were not directly examined in this study, the distal-colon findings suggest that L-fucose-mediated neuroprotection may involve both central and peripheral immune regulation. Further studies examining microbiota-associated glycan remodeling during chronic stress exposure will be important for clarifying the potential involvement of gut–brain interactions in L-fucose-mediated neuroprotection.

Finally, it is worth noting that our molecular analyses focused primarily on the hippocampus, which is not a CSDS-specific brain region. The hippocampus is highly sensitive to chronic stress and closely associated with neuroinflammation and synaptic plasticity. CSDS affects multiple interconnected stress- and emotion-related regions, including the nucleus accumbens (NAc) and ventral tegmental area (VTA) loop, medial prefrontal cortex, amygdala, and hippocampus (19, 20, 75–77). Therefore, future studies examining additional CSDS-relevant brain regions will be needed to determine whether impaired GDP-fucose biosynthesis and core fucosylation are hippocampus-predominant changes or broader circuit-level alterations induced by chronic psychosocial stress.

## Conclusion

In summary, this study provides new evidence that chronic psychosocial stress is associated with disrupted glycometabolic homeostasis, including reduced GDP-fucose availability, decreased expression of key enzymes in the GDP-fucose biosynthetic salvage pathway, and impaired core fucosylation. These alterations are accompanied by increased microglial accumulation, enhanced JAK2/STAT3 inflammatory signaling, reduced expression of synaptic proteins, and depressive-like behaviors, all of which are partially attenuated by exogenous L-fucose. Our findings further suggest that targeting fucose metabolic activation may be a promising therapeutic strategy for stress-related neuropsychiatric disorders.

## Data Availability

The original contributions presented in the study are included in the article/supplementary material. Further inquiries can be directed to the corresponding authors.
