# Supplementary material for: L-fucose administration ameliorates chronic social defeat stress-induced depressive-like behaviors by restoring GDP-fucose biosynthetic activation and core fucosylation
Source: Front Immunol. 2026 Jul 30;17:1909378. doi: 10.3389/fimmu.2026.1909378 (PMC13468815; doi:10.3389/fimmu.2026.1909378)
Supplement: Supplementary file 1 [file DataSheet1.pdf]

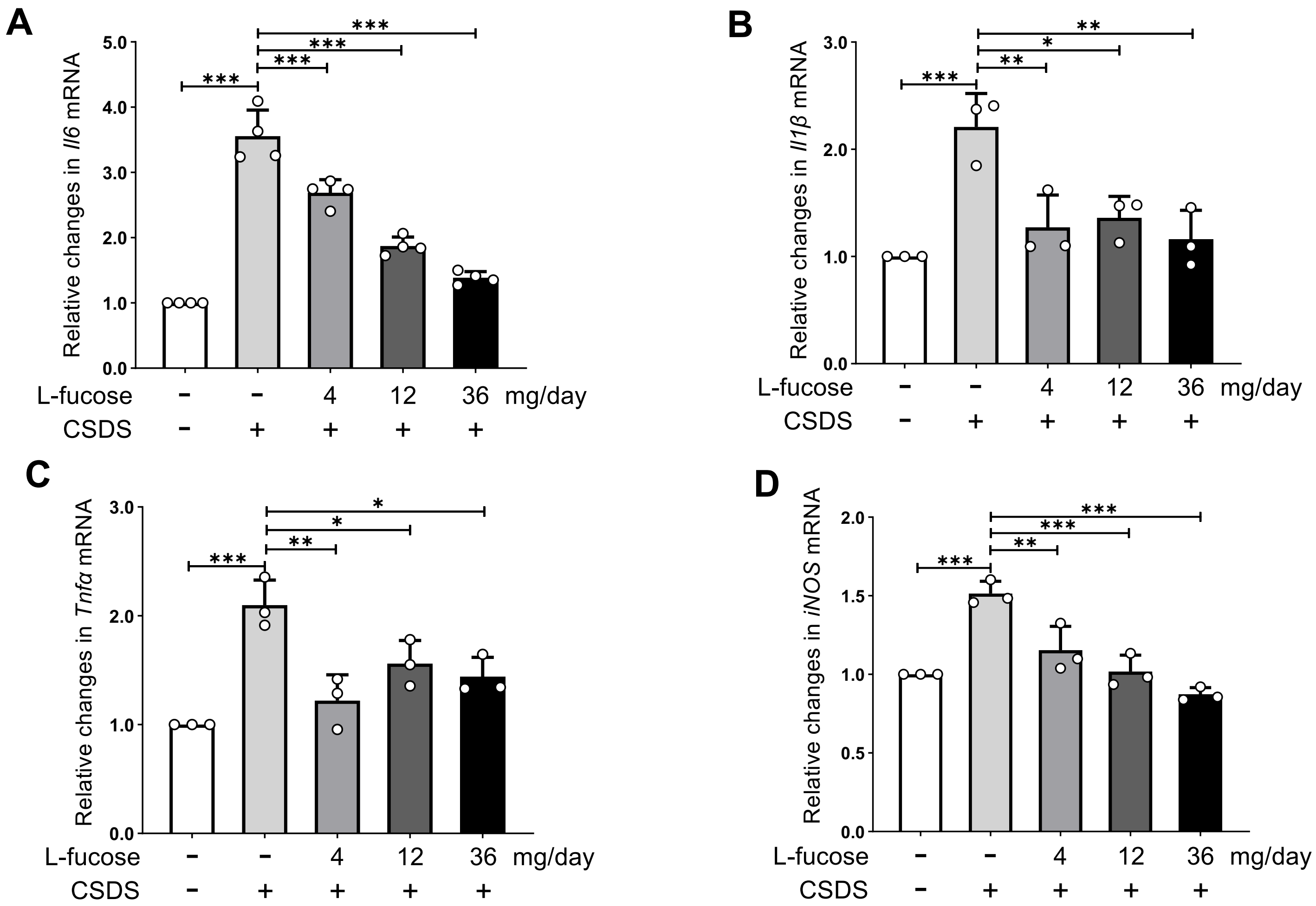

Figure S1. L-fucose attenuated CSDS-induced neuroinflammation. Inflammatory gene expression in hippocampal tissues was assessed by qPCR to evaluate the effects of L-fucose administered at the indicated doses (4, 12, or 36 mg/day) twice daily for 14 days. Relative expression changes in (A) interleukin-6 (*Il6*), (B) interleukin 1β (*Il1β*), (C) tumor necrosis factor-alpha (*Tnfα*), and (D) inducible nitric oxide synthase (*iNOS*) were normalized to *Gapdh*. The ratio in *Fut8*$^{+/+}$ mice without CSDS and L-fucose administration was set to 1.0. All data are presented as mean ± SD (n = 3-4 mice per group). Statistical analysis was performed using one-way ANOVA with Tukey's multiple-comparison test. ns, $p > 0.05$; *$p < 0.05$; **$p < 0.01$; ***$p < 0.001$.

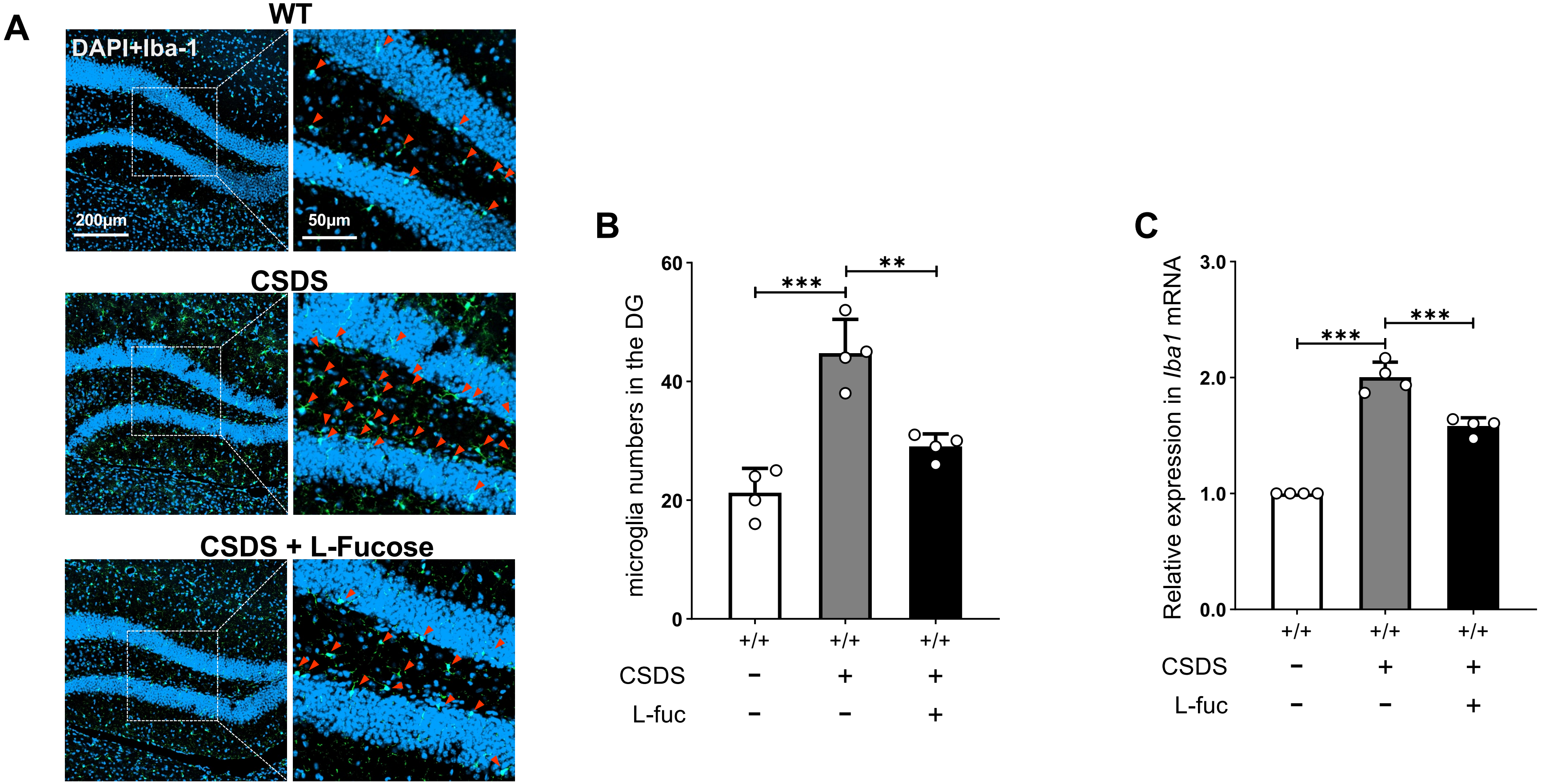

Figure S2. L-fucose attenuates CSDS-induced microglial activation in the hippocampal dentate gyrus (DG). (A) Representative immunofluorescence images of Iba1-positive microglia in the DG. Enlarged images of the boxed regions are shown in the right panels. Scale bars: 200 μm (left panels) and 50 μm (right panels). Sections were stained with Iba1 (green) and counterstained with DAPI (blue). (B) Quantification of Iba1–positive microglia in the DG using ImageJ. (C) qPCR analysis of *Iba1* mRNA levels in the DG, normalized to *Gapdh* (internal control). *Fut8*$^{+/+}$ mice without CSDS exposure and L-fucose were set to 1.0. Statistical analyses were conducted using one-way ANOVA with Tukey's multiple-comparison test. All data are presented as mean ± SD (n = 4 per group). $^{**}p < 0.01$; $^{***}p < 0.001$.

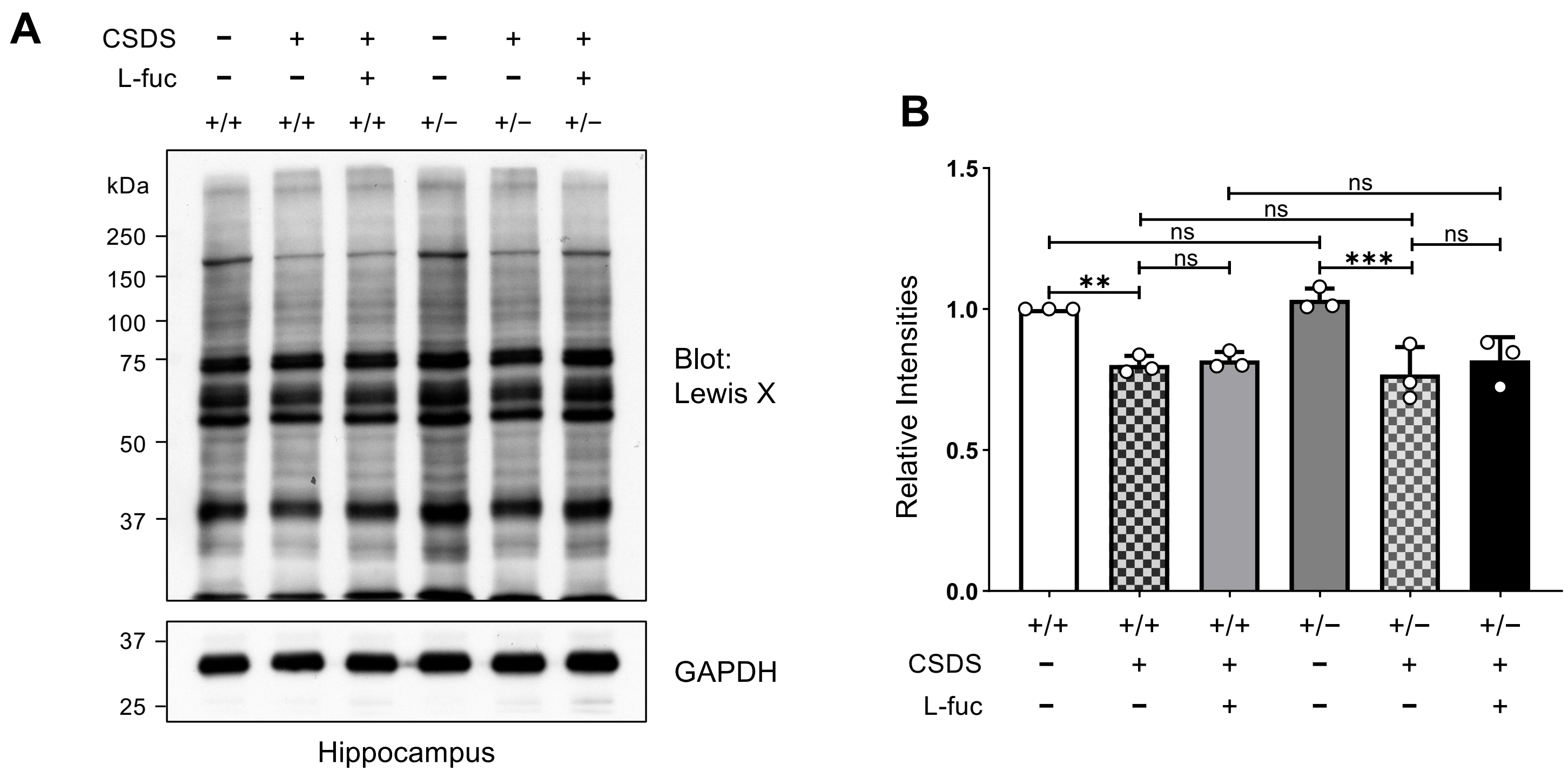

Figure S3. Effects of CSDS and L-fucose treatment on Lewis X expression in the hippocampus of mice. (A) Lewis X expression in hippocampal tissues from Fut8$^{+/+}$ and Fut8$^{+/-}$ mice after CSDS exposure and L-fucose administration was assessed by Western blot with an anti-Lewis X antibody. GAPDH served as a loading control. (B) Quantification of Lewis X expression normalized to GAPDH. Ratios of Lewis X to GAPDH in Fut8$^{+/+}$ mice without CSDS exposure and L-fucose were set to 1.0. Statistical analyses were performed using two-way ANOVA followed by Tukey's multiple-comparison test. All data are presented as mean ± SD (n = 3 mice per group). ns, $p > 0.05$; ** $p < 0.01$; *** $p < 0.001$.
